# A Survey of Dense Low Energy Ions in Earth's Outer Magnetosphere: Relation to Solar Wind Dynamic Pressure, IMF, and Magnetospheric Activity

**DOI:** 10.1029/2021JA029208

**Published:** 2021-09-03

**Authors:** Arthur J. Hull, Oleksiy Agapitov, Forrest S. Mozer, James P. McFadden, Vassilis Angelopoulos

**Affiliations:** ^1^ Space Sciences Laboratory University of California, Berkeley Berkeley CA USA; ^2^ Earth, Planetary and Space Sciences Institute of Geophysics and Space Physics University of California, Los Angeles Los Angeles CA USA

**Keywords:** plasmaspheric plumes, warm plasma cloak, ion outflow, cold dense magnetospheric plasma, low energy ions, outer magnetosphere

## Abstract

The properties of cold, dense, low energy (<150 eV) ions within Earth's magnetosphere between 6 and 14 $R_{E}$ distance are examined using data sampled by Time History of Events and Macroscale Interactions during Substorms spacecraft during a new low‐energy plasma mode that operated from June 2016 to July 2017. These ions are a persistent feature of the magnetosphere during enhanced solar wind dynamic pressure and/or magnetospheric activity. These ions have densities ranging from 0.5 to tens of ${cm}^{-3}$, with a mean of ∼1 ${cm}^{-3}$ and temperatures of a few to tens of eV, with a mean of ∼13 eV. These yield cold to hot ion density and temperature ratios that are 4.4 and $4\times 10^{-3}$, respectively. Comparisons reveal that the cold ion densities are positively correlated with solar wind dynamic pressure. These ions are organizable, according to their pitch‐angle distribution, as being transverse/convection dominated (interpreted as plume plasma) or magnetic field‐aligned (FAL) (uni‐ or bi‐directional characteristic of ion outflow or cloak plasma). Transverse ions preferentially occur in the prenoon to dusk sectors during sustained active magnetospheric conditions driven by enhanced solar wind dynamic pressure under southward $B_{z}$ and westward $B_{y}$ IMF orientations. Transverse ion velocities (reaching several tens of km/s) have a westward directed tendency with a slight radially outward preference. In contrast FAL ions preferentially occur from morning to noon during northward IMF orientations, enhanced solar wind dynamic pressure, and quiet magnetospheric conditions within several hours after moderate to strong activity. The FAL ions also have bulk velocities ≲30 km/s, with an eastward and radially outward tendency.

## Key Points

- Dense low energy ions are a persistent feature of the magnetosphere during enhanced solar wind dynamic pressure and/or *Kp*
- Low energy transverse ions tend to occur in duskside magnetosphere under southward *B*_*z*_ and westward *B*_*y*_ IMF conditions, with westward flow
- Low energy magnetic field‐aligned (uni‐ or bi‐directional) ions tend to occur in the dawnside under northward IMF, with an eastward flow

## 1 Introduction

In addition to hot energetic ions that make up the plasma sheet and ring current populations, the magnetosphere often contains significant populations of low energy ions. Though they do not contribute much to magnetospheric pressure, the low energy ion constituents usually provide the dominant contribution to plasma density when they occur in the magnetosphere. Sources of low energy ions in the magnetosphere stem from plasmaspheric plumes or ionospheric ion outflows.

Plasmaspheric plume ions have origins from the plasmasphere, which is composed of plasma from the ionosphere trapped by the Earth's corotational electric field. During geomagnetically active times, some of the plasmaspheric ion population at the outer edges break away and subsequently penetrate to different parts of the dayside magnetosphere owing to their enhanced E×B drifts attributed to the enhancement of the dawn‐dusk electric field (e.g., Chappell, 1974; Elphic et al., 1996; Goldstein, 2006; Darrouzet et al., 2009, and references therein). This population manifests as plumes of ejecta that usually extend from the duskside outer plasmasphere into the magnetosphere, and often all the way out to the magnetopause (McFadden et al., 2008b; Sauvaud et al., 2001), as these ions drift radially outward and westward. The plume ions have energies near a few eV and below, but can occur at higher energies in association with enhanced convection in the magnetosphere, or in the vicinity of magnetopause in association with magnetopause motion and/or enhanced reconnection flows (Chen & Moore, 2004; McFadden et al., 2008b; Sauvaud et al., 2001). Plume occurrences in the vicinity of the magnetopause have also been shown to be associated with enhanced solar wind dynamic pressure (e.g., Lee et al., 2016). Plumes can impact outer radiation belt electron dynamics. This is due to the fact that plumes are associated with sharp density gradients that facilitate the entry of chorus waves into the plasmasphere, where they can seed the production of plasmaspheric hiss emissions (Agapitov et al., 2018; Hartley et al., 2019), and as a consequence result in conditions that give rise to efficient scattering of radiation belt electrons by whistler waves (Summers et al., 2008).

Ionospheric outflows present another important source of low energy ions in the equatorial magnetosphere. These may be from polar wind ions, and low energy ion outflows from the auroral and cusp regions. Polar wind ions are an ambipolar outflow along the field at latitudes above the plasmasphere (e.g., Yau et al., 2007). Outflows from the auroral regions and cusp may be from upwelling ions, ion conics, or ion beams (e.g., Shelley, 1995; Welling et al., 2015). Ion beams, which are the result of energization in quasi‐static parallel electric fields, are field‐aligned and have been observed at energies from a few tens of eV to a few keV. Both upwelling ions and ion conics are upgoing ions that are the result of transverse ion acceleration at lower altitudes and have peak fluxes at pitch‐angles that are oriented somewhat off from the magnetic field direction. In the equatorial plane these become field‐aligned, owing to the folding effect of the magnetic mirror force and are difficult to distinguish from ion beams. Ionospheric ions in the equatorial plane have been observed with either unidirectional or bidirectional magnetic field‐aligned fluxes. Unidirectional ion outflows are directly transported to the magnetosphere and primarily occur in the nightside (e.g., Nagai et al., 1983) during enhanced geomagnetic activity, such as from geomagnetic storms and substorms (e.g., Gkioulidou et al., 2019; Hull et al., 2019). Bidirectional ions are the result of being trapped on closed field lines or are outflowing ions from opposing northern and southern hemispheres. A well‐known example of bidirectional ionospheric outflow sourced plasma commonly observed in the magnetosphere is the warm ion cloak, which has been the focus of numerous past studies from a variety of in‐situ satellites (e.g., Borovsky et al., 2013; Chappell et al., 1987, 2008; DeForest & McIlwain, 1971; Fennell et al., 1981; Fuselier et al., 2017; Horita et al., 1987; Nagai et al., 1983; Sagawa et al., 1987, and references therein). This ion population is composed of magnetically trapped counterstreaming ions at energies from a few to a few 100 eV that predominantly occur in the dawn side of the magnetosphere. These ions can also extend out to the magnetopause where they can impact reconnection processes (e.g., Borovsky et al., 2013; Fuselier et al., 2017). Trajectory analysis of ions under steady southward IMF conditions suggest that the warm plasma cloak ions can originate from polar wind ions that convect over the polar cap and into nightside magnetotail at distances 8–45 $R_{E}$ before drifting back toward Earth to form a cloak of bouncing ions that extends from the nightside magnetosphere into the morning sector and postnoon depending on energy (Chappell et al., 1987). Additional contributions may also stem from transverse energized ion outflow from the auroral region, particularly during quieter periods.

Other examples of ion outflow that appear distinct from warm ion cloak ions have been observed in the vicinity of the equatorial magnetopause (e.g., Fuselier et al., 2019; Lee et al., 2016). In the report by Lee et al. (2016) ion outflows were indicated by counterstreaming energy dispersed ion signatures with energies (ranging from a few eV to keV) that decreased with decreasing L values away from the magnetopause current layer. In addition McFadden et al. (2008a) reported, Time History of Events and Macroscale Interactions during Substorms (THEMIS) observations of an extended interval (∼1 h) of low energy (5–200 eV) field‐aligned ions in the postnoon sector traveling inward from the magnetopause.

Though significant progress has been made, the properties, distribution, and factors that control the occurrence of cold dense plasma in the magnetosphere are not fully understood. A more complete understanding of the dense low energy plasma in the magnetosphere is essential since such ions can impact among other things the magnetosphere structure and dynamics, reconnection processes at the dayside magnetopause (e.g., André et al., 2016; Borovsky et al., 2013; Fuselier et al., 2017; Walsh et al., 2014), and also the growth rates of waves (e.g., whistler chorus and hiss, and electromagnetic ion cyclotron waves) and hence their distribution and ability to participate in energization and loss processes in the magnetosphere (e.g., Li et al., 2007; Turner et al., 2014). Moreover, the low energy plasma populations may also act as a seed population for energetic plasma populations through subsequent energization processes.

This study is focused on the statistical properties of dense low energy ions within Earth's magnetosphere at distances from 6 $R_{E}$ to 12 $R_{E}$ using data from the THEMIS A and D spacecraft in the interval spanning June 22, 2016 to August 31, 2017. During most of this period, the THEMIS A and D sampled electrons and ions in the magnetosphere in a cold plasma mode, which was optimized to sample plasma entering the electrostatic analyzers at low energies (0–25 eV) with unprecedented energy resolution. This mode provides a better assessment of lower energy electron and ion populations than other periods of the THEMIS mission, which was limited to 7 eV energies and above. In addition the apogees of the THEMIS A and D spacecraft (14 and 12.4 $R_{E}$, respectively) are also suited to sample low energy ions over a large spatial extent of the equatorial magnetosphere out to the magnetopause boundary where these ions can impact reconnection processes. Our approach is comprehensive in that we examine the properties of the low energy ions as a composite and also based on their flux characteristics, which are distinguished as being transverse/convective dominated or field‐aligned (uni‐ or bi‐directional) ions. This approach is similar to that taken by Lee et al. (2016), who examined Cluster observations of a relatively limited sample of cold plasma observations near the magnetopause proper. In this study, we present the properties of low energy ion populations in case and statistical analysis including their spatial distribution, moment properties (density, temperature, and flow), and relation to solar wind driving conditions and magnetospheric activity measures.

## 2 Instrumentation, Experimental Data Set, and Event Selection

This study is primarily based on plasma and field data from the THEMIS A and D spacecraft sampled in the magnetosphere during cold plasma mode, which was operational in the interval spanning June 21, 2016 to June 27, 2017. During this period THEMIS‐D was in cold plasma mode from June 21, 2016 to October 23, 2016 and THEMIS‐A over the interval spanning October 24, 2016 to June 27, 2017. Additional data spanning from June 28, 2017 to August 31, 2017 is included in the statistical component of this study to provide more complete coverage of data in the ecliptic plane magnetosphere. The plasma data are from the THEMIS plasma instrument (McFadden, Carlson, Larson, Ludlam, et al., 2008), which is composed of a pair of top hat electrostatic analyzers (ESAs), one for ions and one for electrons. During the cold plasma mode, the energy sampling for the THEMIS ESAs were optimized to sample low energy ions and electrons with 15 nearly equal energy steps being used from 0 to 25 eV and with 18 increasing energy steps from 25 eV to ∼30 keV. To assess the properties of cold and hot ion plasma components partial moments of spin period resolution (3s) full three‐dimensional (3D) ion velocity distributions were computed in the energy range from 0 to 150 eV for cold ions and 150 eV to ∼30 keV for hot ions after correcting these ranges for the spacecraft floating potential measured by THEMIS‐electric field instrument (Bonnell et al., 2008). To select data corresponding to magnetospheric ions, we required the temperature of hot electrons (≥25 eV energies above the spacecraft potential) to be >100 eV. Given that magnetosheath electrons typically have post‐shocked temperature values of up to several tens of eV, this criteria is quite effective in discriminating intervals in the magnetosheath plasma from those in the magnetosphere (Walsh et al., 2020).

NASA OMNIWeb solar wind parameters (e.g., IMF, and dynamic pressure) mapped to the bow shock nose and magnetospheric activity measures (e.g., Kp) were also used in this study to examine dependencies on solar wind driving conditions and magnetospheric activity.

## 3 Case Studies

### 3.1 June 22, 2016 Event

**Figure 1:**
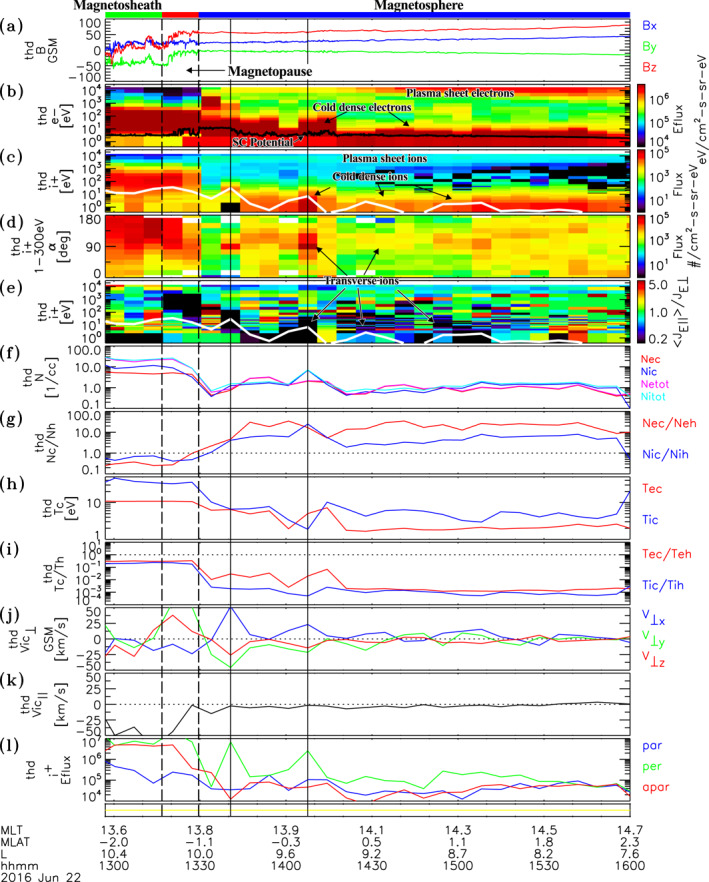
Plasma and field data from Time History of Events and Macroscale Interactions during Substorms D observed on June 22, 2016. Shows (a) Magnetic field in GSM coordinates, (b) Electron energy flux versus energy, (c) Ion flux versus energy, (d) Spectra of flux versus pitch‐angle for 1–300 eV ions, (e) Ratio of parallel to perpendicular ion fluxes versus energy, (f) Densities, (g) Density ratios, (h) Cold electron (red) and ion (blue) temperatures, (i) Temperature ratios of cold and hot electrons (red) and ions (blue), ion velocities (j) Perpendicular and (k) Parallel to the background magnetic field $B_{o}$, (l) Peak energy fluxes of ions parallel (blue), perpendicular (green) and antiparallel (red) to $B_{o}$. The black curve in panel (b) is the spacecraft potential. The white curve in panels (c) and (e) give the ion bulk energy. The yellow bar at the bottom indicates data were sampled in slow survey mode. The dashed vertical lines delimit the magnetopause.

An example of cold dense plasma observations is given in Figure 1, which shows plasma and field data sampled during an inbound transect from the magnetosheath, across the magnetopause current layer, and into the dayside magnetosphere (near 14 MLT) by THEMIS D on June 22, 2016. During the event, the 3 h Kp index transitioned from $2^{-}$ to $3^{+}$. The IMF was preferentially oriented in the westward direction with a median $IMF\;B_{y_{GSM}}=-6.0$ nT, and $IMF\;B_{z_{GSM}}=-1.3$ nT over the cold plasma interval. The dynamic solar wind pressure was also moderately elevated with values $P_{\text{SW}}∼2-3\;nPa$. The dashed vertical lines at ∼1318 UT and ∼1330 UT delimit the magnetopause current layer, which marks the transition from highly variable magnetosheath magnetic fields to a dipole‐like magnetic field configuration characteristic of the magnetosphere (see Figure 1a). The magnetopause current layer, magnetosheath, and magnetosphere regions are indicated by the red, green, and blue bars at the top of Figure 1, respectively. In the magnetosphere, just after the magnetopause current layer (i.e., after ∼1330 UT), two plasma populations are observed. One population corresponds to the plasma sheet, which are the energetic electrons (yellow to red) and ions (cyan to green) at a few keV energies and above in the omnidirectional electron energy flux and ion flux spectra shown in Figures 1b and 1c, respectively. Note that the yellow bar at the bottom of Figure 1 indicates that the plasma data were sampled in slow survey mode, during which the full 3D electron and ion distribution measurements and thus the energy spectra and moments shown are provided at spin period resolution (3s) every 6.7 min in the interval. In addition there is also a low energy plasma population that is distinct from the plasma sheet. The low energy electron component is indicated by the red signatures at and up to a few tens of eV above the black spacecraft potential curve in the electron energy flux spectra shown in Figure 1b. This population is in the energy range of low energy electrons reported in the recent studies (McFadden et al., 2008b; Mozer et al., 2017; Walsh et al., 2020). The corresponding low energy ion component is indicated by the yellow to red signatures at tens of eV and below in the ion flux spectra depicted in Figure 1c.

Figure 1f depicts the densities of low energy electrons (red) and ions (blue) in addition to the total densities of electrons (magenta) and ions (cyan) over the ESA detector energy range. The low energy electrons and ions are relatively dense having densities ∼1–2 ${cm}^{-3}$ over much of the interval with values reaching ∼5–6 ${cm}^{-3}$ closer to the magnetopause. This low energy population provides the dominant contribution to density, as evidenced by the nearly matching low energy and total densities of electrons and ions in Figure 1f. Figure 1g shows that the ratio between cold and hot densities for ions and electrons are quite large reaching 20–30 in places. The reasonably close agreement between the electron and ion densities indicates that the cold electron and ion populations are well measured in the low energy sampling mode of the ESAs and that the ions are consistent with being predominantly composed of protons. Note, Poisson statistical uncertainties and up to ±1 Volt uncertainties in the spacecraft potential (Mozer et al., 2017) give rise to uncertainties in the cold ion densities estimated at $\delta N_{ic}/N_{ic}\;≲$ 20%. The Poisson uncertainty for cold electrons is negligible, owing to their much higher count rate. However, uncertainties in electron densities owing to ±1 Volt uncertainties in the spacecraft potential are non‐negligible and are estimated at $\delta N_{ec}/N_{ec}\;∼$ 10%–25% for electrons with 5–8 eV temperatures and up to $\delta N_{ec}/N_{ec}\;∼$ 40%–70% for the coldest electrons (∼2 eV) in the interval. The low energy electrons and ions are quite cold with temperatures ranging from a few to 10 eV for both constituents (see Figure 1h). In contrast, plasma sheet electrons and ions have temperatures of ∼1–2 keV and 5–8 keV, respectively (not shown), which yield ratios of cold and hot temperatures for electrons (blue) and ions (ions) shown in Figure 1i reaching as low as 0.001 and 0.0005, respectively. Note that uncertainties in the temperature of the cold dense ion population due to Poisson statistics and spacecraft potential are estimated at $\delta T_{ic}/T_{ic}≲$ 20%, whereas the cold dense electron temperature uncertainties are typically $\delta T_{ec}/T_{ec}\;∼10\%$ (mostly from the spacecraft potential uncertainty). In addition the finite energy steps in the cold plasma sampling mode place a limit (∼1.5 eV) on the smallest resolvable temperature by the THEMIS ESA detectors. The electron and ion temperatures near this limit should be considered as rough order of magnitude estimates.

Figures 1j and 1k show the velocities perpendicular (in GSM coordinates) and parallel to the magnetic field, respectively. With some exceptions, the low energy ions are primarily moving transverse to the magnetic field and toward the magnetopause, as indicated by the much larger variations in the perpendicular velocity with little to no parallel velocity component. The magnitude of the cold ion perpendicular velocity shows variations that are largest closer to the magnetopause, having a peak value reaching 76 km/s at the first solid vertical line, but reduces to ∼10 km/s (or less) at the end of the cold dense ion interval, as the spacecraft moves deeper into the magnetosphere to lower L‐shells. Figure 1l shows peak energy fluxes of low energy ions (at energies below 300 eV) parallel (blue), perpendicular (green), and antiparallel (red) to the magnetic field. Consistent with the flow characteristics, the low energy ions have energy fluxes in the spacecraft frame that are strongly peaked perpendicular to the magnetic field, with significantly lower energy fluxes in the parallel or antiparallel direction throughout most of the interval where they occur. Associated with the enhanced densities and perpendicular flows, the energy flux is seen to increase significantly, as the cold plasma population approaches the magnetopause. This is also demonstrated in Figure 1e, which shows the spectrogram of the ion anisotropy measured as the ratio between the average of the parallel and antiparallel fluxes divided by the perpendicular fluxes. The low energy ions with preferential perpendicular anisotropy are indicated by the blue to black signatures. To compare, the white curve in Figure 1e indicates the cold ion bulk flow energy, which shows variations that correspond to energies of enhanced preferential perpendicular anisotropies. The transverse nature is also revealed by the preferential enhancements near 90° in the pitch‐angle spectra of ions at energies from 1 to 300 eV in Figure 1d. The observed preferential transverse fluxes and perpendicular flow indicate that these ions are strongly controlled by convection. These signatures indicate that these ions are plasmaspheric plume ions.

**Figure 2:**
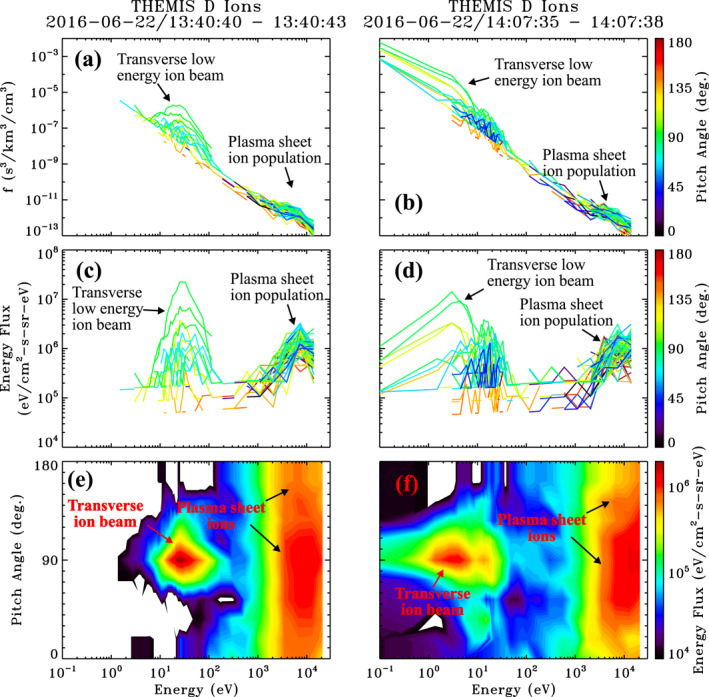
Example 3s resolution ion distributions from the June 22, 2016 event sampled at the two times indicated by the solid vertical lines in Figure 1. Shown are (a and b) Ion phase space density and (c and d) Differential energy flux spectra as a function of energy for different pitch‐angles, and (e and f) Contour plots of differential energy flux as a function of energy and pitch‐angle for the times listed above each column.

Figures 2a and 2b show examples of 3s resolution phase space density as a function of energy of ions sampled at 1340:40 UT and 1407:35 UT, respectively, indicated by the solid black vertical lines in Figure 1. Corresponding differential energy flux spectra are given in Figures 2c and 2d. The distribution and energy flux spectra are color coded according to pitch‐angle with respect to the spin averaged background magnetic field. Contour plots of differential energy flux as a function of energy and pitch‐angle are also given in Figures 2e and 2f for the two time samples. The phase space and energy flux distributions in the first example show a prominent peak near 25 eV at a pitch‐angle of 90° (green lines in Figures 2a and 2c, red enhancement in Figure 2e), with no apparent signature at other pitch‐angles. This is a cold dense ion population with a density of $N_{\text{ic}}=1.3\;{cm}^{-3}$ and temperature of $T_{\text{ic}}=7$ eV. A second population with a slight preferential perpendicular anisotropy is seen at keV energies attributed to the plasma sheet ion population. This population is more apparent in the energy flux spectra and contour plots depicted in Figures 2c and 2e (yellow to red enhancement spanning 0°–180° pitch‐angles), respectively. In this case, the plasma sheet ion population has a density of $N_{\text{ih}}=0.3\;{cm}^{-3}$ and temperature of $T_{\text{ih}}=3500$ eV. The second event displayed in Figures 2b, 2d and 2f were sampled 27 min later and illustrates the importance of the low energy sampling mode. It shows a low energy ion population near and below a few eV energies. This population is denser and colder than the previous example, with $N_{\text{ic}}=5\;{cm}^{-3}$ and $T_{\text{ic}}=2.0$ eV. A plasma sheet ion population with a slight preferential perpendicular anisotropy is again observed above 1 keV energies, with a respective density and temperature estimated at $N_{\text{ih}}=0.3\;{cm}^{-3}$ and $T_{\text{ih}}=3700$ eV.

### 3.2 June 23, 2016 Event

**Figure 3:**
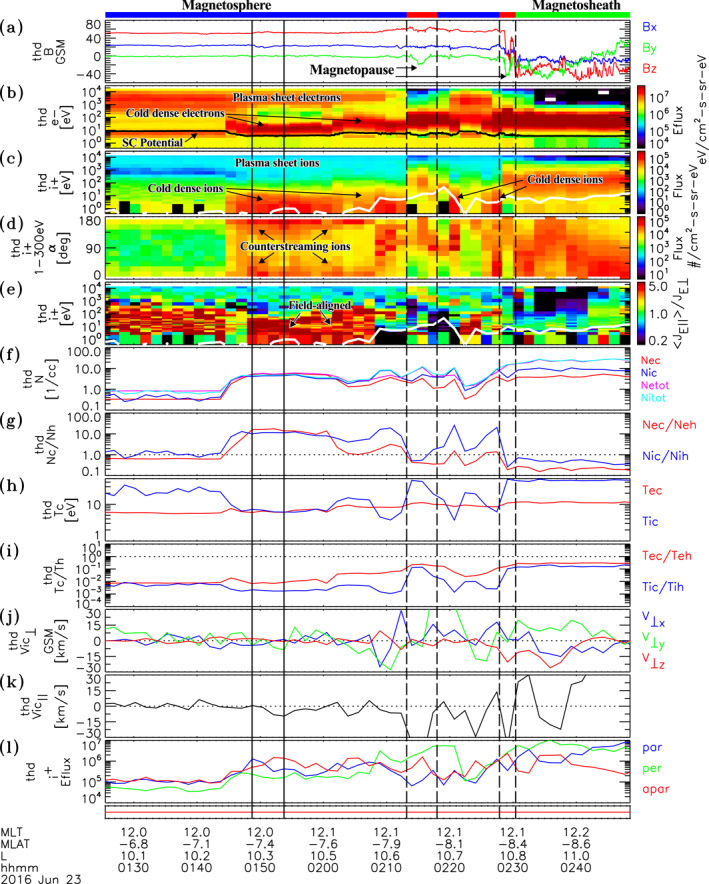
Plasma and field data from Time History of Events and Macroscale Interactions during Substorms D observed on June 23, 2016. Same format as Figure 1. The red bar at bottom indicates that the data were sampled during fast survey mode. The two sets of dashed vertical lines delimit a partial and full crossing of the magnetopause, respectively.

Another example of cold dense plasma observations is given in Figure 3, which shows plasma and field data sampled during an outbound transect of the magnetosphere, followed by subsequent in‐out crossings of the magnetopause into the magnetosheath by THEMIS D on June 23, 2016. This event occurred during relatively quiet magnetospheric conditions (Kp=2) and under steady northward IMF conditions with $IMF\;B_{y_{GSM}}=-0.05$ nT, and $IMF\;B_{z_{GSM}}=2.19$ nT. The dynamic solar wind pressure was also moderately elevated with $P_{\text{SW}}∼2\;nPa$. The red bars at the top of Figure 3 indicate a partial and subsequent full crossing of the magnetopause current layer, while the green and blue bars indicate the magnetosheath and magnetosphere sides. Note that the red bar at the bottom of Figure 3 indicates that the plasma data were sampled in fast survey mode, during which the full 3D electron and ion distribution measurements and thus the energy spectra and moments shown are provided at spin period resolution every ∼1.5 min in the interval. Dense low energy electrons (tens of eV above the spacecraft potential line in Figure 3b) and ions (≲20–30 eV in Figure 3c) are observed from ∼0145:23 to just prior to the first partial crossing of the magnetopause at 0213:05 UT and also within the partial reentry of the magnetosphere in the interval from 0218:00 UT to 0226:37 UT. The matching cold electron and ion densities where these occur indicate that the ions are well measured and are consistent with being primarily composed of protons. This population has density ranging from $N_{\text{ic}}∼\;2$ to ∼8 ${cm}^{-3}$, with cold to hot density ratios approaching $N_{\text{ic}}/N_{\text{ih}}∼$ 10–18 (see Figures 3f and 3g). The cold ion temperatures are found to range from $T_{\text{ic}}∼4$ to ∼15 eV, yielding cold to hot ion temperature ratios as low as $T_{\text{ic}}/T_{\text{ih}}∼10^{-3}$ (see Figures 3h and 3i, respectively). The combined Poisson and spacecraft potential uncertainties in the density and temperatures of these cold dense ions are estimated at $\delta N_{ic}/N_{ic}≲10\%$ and $\delta T_{ic}/T_{ic}≲10\%$, respectively. Similarly, the cold dense electron density and temperature uncertainties are estimated at $\delta N_{ec}/N_{ec}≲10\%$ and $\delta T_{ec}/T_{ec}∼5\%$, respectively.

The cold dense ions are preferentially field‐aligned (either unidirectional or counterstreaming depending on location) in the interval from ∼0144:40 UT to ∼0208:00 UT. This is indicated by the red signatures near 0° and 180° in the differential flux pitch‐angle spectra shown in Figure 3d and also the red signatures in the parallel to perpendicular flux ratio spectra at tens of eV and below in Figure 3e. The field‐aligned nature is also assessable from Figure 3l, which shows peak parallel and/or antiparallel energy fluxes that are significantly larger (up to a factor of 8–10) than the perpendicular counterpart in the interval. Subsequent to this and prior to the magnetopause current layer boundary at 0213:05 UT, the low energy ion measurements appear to be admixtures of field‐aligned and perpendicular components.

**Figure 4:**
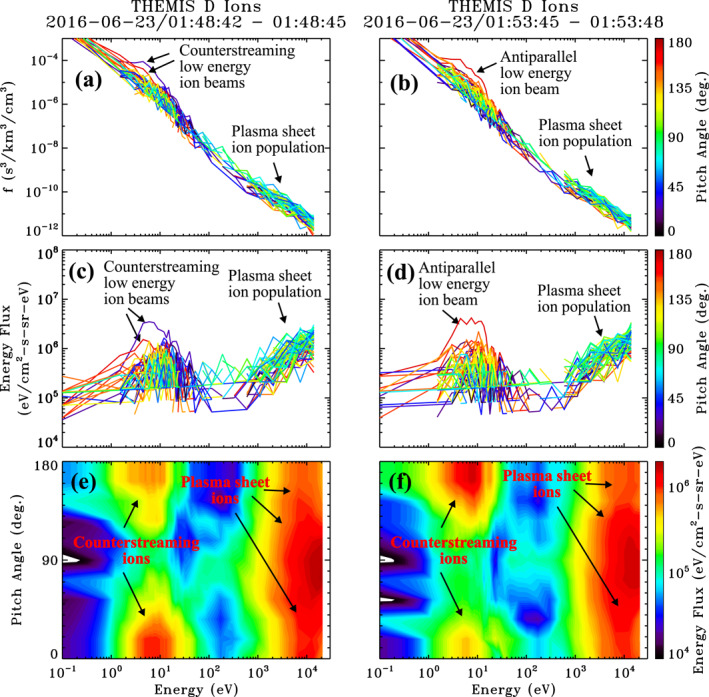
Example 3s resolution ion distributions from the June 23, 2016 event sampled at the two times indicated by the solid vertical lines in Figure 3. Shown are (a and b) Ion phase space density and (c and d) Differential energy flux spectra as a function of energy for different pitch‐angles, and (e and f) contour plots of differential energy flux as a function of energy and pitch‐angle for the times listed above each column.

Ion phase space distribution functions sampled at 0148:42 UT and 0153:45 UT are shown in Figures 4a and 4b, respectively, and indicated by the vertical solid lines in Figure 3. Also spectra (Figures 4c and 4d) and contour plots (Figures 4e and 4f) are shown of differential energy flux for each of these times. The phase space density and energy flux distributions of the first example (Figures 4a, 4c and 4e) reveal the presence of counterstreaming field‐aligned ion beams extending from ∼2 to ∼30 eV energies with a preferential skew parallel (blue) to the background magnetic field. In contrast, the second example, sampled ∼5 min later, shows counterstreaming ions extending from ∼2 to ∼30 eV energies with a pronounced antiparallel sense (red line in Figures 4b and 4d). Though difficult to discern from the line plots, the counterstreaming character is apparent in the contour plot in Figure 4f, which shows clear parallel (yellow to orange) and antiparallel (red) enhancements within the aforementioned energy range. There is no indication of a magnetosheath ion population in both distributions shown in Figure 4, but there is a hot plasma sheet ion population demonstrating that these are on closed field lines in the magnetosphere. Close inspection of electron distributions (not shown) also revealed the presence of cold low energy and hot plasma sheet components with no magnetosheath component, verifying that these are on closed magnetospheric field lines and not within an extended lower latitude boundary layer (e.g., Øieroset et al., 2015). Additional alternating changes in the skew of counterstreaming ion beams are apparent prior to encountering the magnetopause at 0213:05 UT in the pitch‐angle spectra shown in Figure 3d and also in the parallel (blue) and antiparallel (red) peak energy fluxes of low energy ions shown in Figure 3l. These counterstreaming ions do not appear to be attributed to cloak ions, which are observed with lower, symmetrically balanced parallel and antiparallel flux levels from 10 eV to a few hundred eV at the beginning of the interval to roughly ∼0144:40 UT (see Figures 3c–3e and 3l). Without a particle trajectory analysis, which is beyond the scope of this study, it is difficult to know the exact source of these counterstreaming/alternating bouncing cold ions on closed magnetic field‐lines adjacent to the magnetopause. A possible explanation is that these ions are low energy ionospheric outflows convectively transported to this region from the dayside auroral zone/cusp proper as may occur from lobe reconnection characteristic of northward IMF conditions (Fuselier et al., 2019; Lee et al., 2016; McFadden et al., 2008a). The dayside auroral zone/cusp proper is known to be associated with the largest outflow fluxes attributed to relatively higher density and lower outflowing energies than other regions.

### 3.3 May 08, 2017 Event

**Figure 5:**
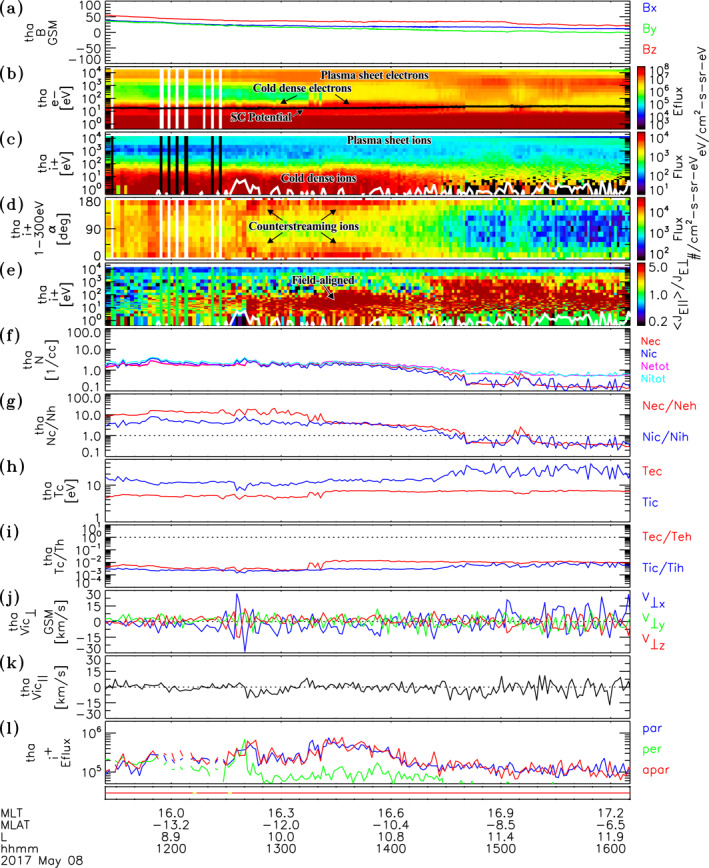
Plasma and field data from Time History of Events and Macroscale Interactions during Substorms A observed on May 08, 2017. Same format as Figure 1. The red bar at bottom indicates that the data were sampled during fast survey mode.

Figure 5 depicts an extended interval of plasma and field data from an outbound traversal of the magnetosphere near dusk (16–16.9 MLT) by THEMIS A on May 08, 2017, during which cold dense ions were observed. This event occurred under relatively quiet magnetospheric conditions ($Kp=1^{-}$), but with moderately elevated dynamic solar wind pressure $P_{\text{SW}}∼3\;nPa$. The IMF was also preferentially northward with $IMF\;B_{y_{GSM}}=1.0$ nT, and $IMF\;B_{z_{GSM}}=3.4$ nT. As indicated by the red bar at the bottom of Figure 5, the plasma distribution and moment data were sampled during fast survey mode, which are at spin period resolution given every ∼1.5 min in the interval shown. Dense low energy electrons and ions are observed from the beginning of the interval to 1440 UT. This region spans a large spatial extent of the magnetosphere from L values of 8.2–11.2. The cold dense electron population corresponds to the enhanced energy fluxes extending up to ∼20 eV above the spacecraft potential indicated by the black curve in Figure 5b. These are collocated with dense low energy ions as indicated by the red enhancements extending up to several tens of eV in the differential flux spectra shown in Figure 5c. This low energy ion population is counterstreaming along the magnetic field, as indicated by the differential flux pitch‐angle spectra shown in Figure 5d. The peak parallel (blue) and antiparallel (red) energy fluxes of these ions in Figure 5l are symmetrically balanced and, for much of the interval, much larger than the transverse counterpart (green). The parallel energies of these ions (near 100 eV and below) are larger than the bulk kinetic energies for these ions indicated by the white solid curve in Figures 5c and 5e. This is owing to the fact that the symmetrically balanced parallel fluxes yield nearly net zero parallel bulk flows (see Figure 5k) and convection is also low in the interval (see Figure 5j). The density of this population ranges from 1 to 4 ${cm}^{-3}$, with a median of 2 ${cm}^{-3}$, yielding median cold to hot density ratios of 9 and 4 for electrons and ions, respectively (see Figures 5f and 5g). The reasonably close agreement between cold electron and ion densities indicates that the ions are predominantly composed of protons. The low energy electron temperatures range from $T_{\text{ec}}∼\;4$ to 7 eV with a median of 5.3 eV, yielding median cold to hot electron temperature ratios of $T_{\text{ec}}/T_{\text{eh}}∼5\times 10^{-3}$ (see Figures 5h and 5i). The low energy ions are relatively warmer with temperatures ranging from $T_{\text{ic}}∼\;7$ to 18 eV with a median of 12 eV, yielding median cold to hot ion temperature ratios $T_{\text{ic}}/T_{\text{ih}}∼3\times 10^{-3}$. The uncertainties in the cold ion densities and temperatures attributed to Poisson and spacecraft potential errors are estimated at $\delta N_{ic}/N_{ic}\;∼9-14\%$ and $\delta T_{ic}/T_{ic}∼7-10\%$, respectively. Similarly, for the electrons $\delta N_{ec}/N_{ec}\;∼10-20\%$ and $\delta T_{ec}/T_{ec}\;∼3\%$. Subsequent to this interval, this population becomes attenuated, as its density contribution reduces to 0.2 ${cm}^{-3}$. The symmetric counterstreaming nature over the large spatial extent of the magnetosphere indicates that these ions have characteristics of warm cloak ions, however these are well into the dusk sector of the magnetosphere as opposed to the dawn sector.

## 4 Statistics

**Figure 6:**
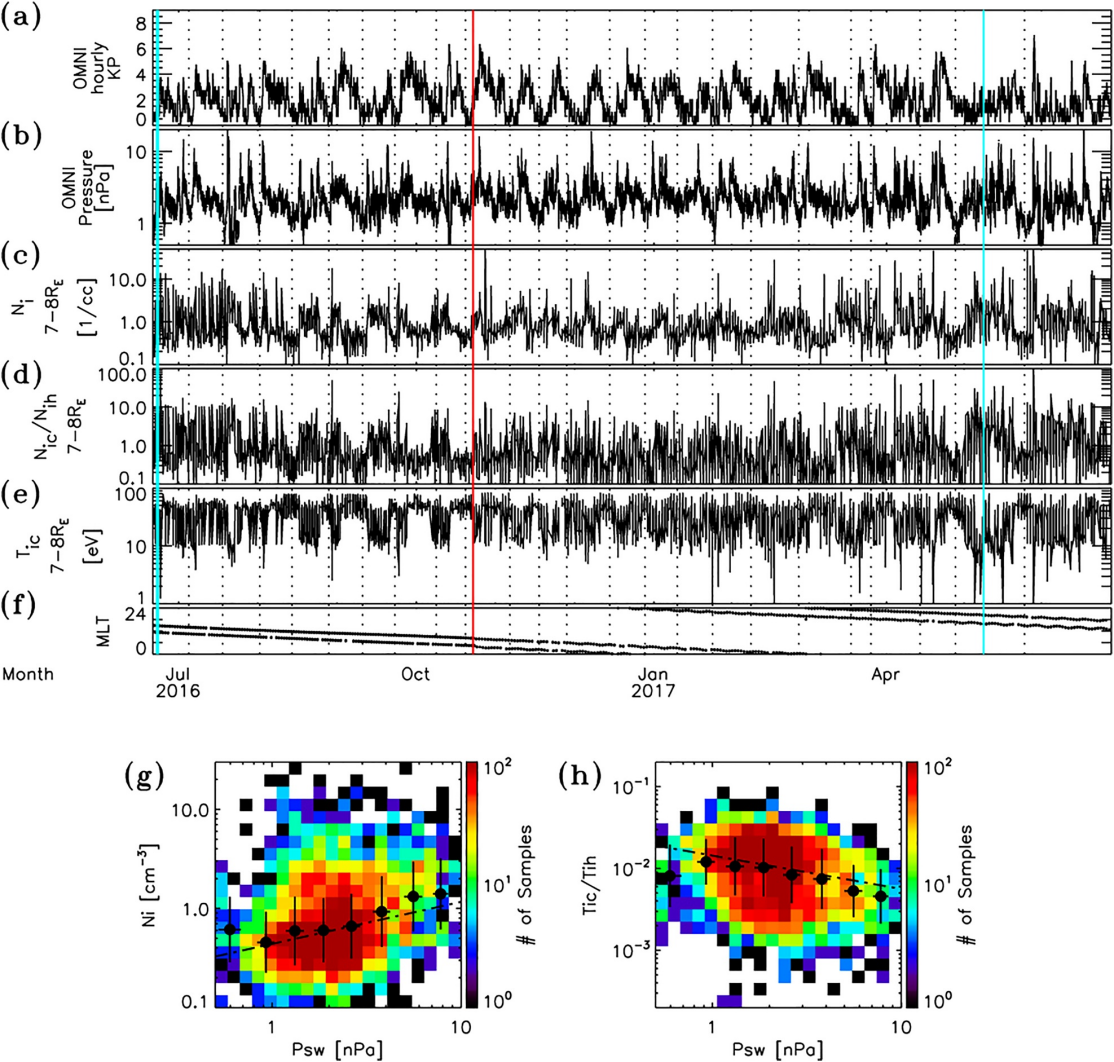
Shows (a) Kp, (b) Solar wind dynamic pressure. Also show (c) Ion density, (d) Cold to hot density ratios, and (e) Cold ion temperatures from Time History of Events and Macroscale Interactions during Substorms (THEMIS) A and D measurements sampled in the magnetosphere at distances from 7 to 8 $R_{E}$ in the interval from June 21, 2016 to June 27, 2017. (f) Inbound and outbound spacecraft magnetic local time locations at these distances. Data prior to the red vertical line are from THEMIS D and after from THEMIS A. The cyan vertical lines indicate the times corresponding to the events described in Section 3. The dotted vertical lines are provided at select pressure minima as a visual aid. The distribution of ion measurements as a function of (g) Ion density and solar pressure $P_{\text{SW}}$, and (h) Cold‐hot ion temperature ratio and $P_{\text{SW}}$. The solid black dots and bars in panels (g) and (h) represent log space bin averages and standard deviations of data samples, while the dash‐dotted lines are linear fits.

To examine the temporal behavior of magnetospheric density in relation to the presence of cold dense ions, magnetospheric activity, and solar wind pressure over an extended period of time, Figure 6 shows roughly 1 year (June 21, 2016 to June 27, 2017) of combined cold plasma mode sampled data from THEMIS A and D at distances ranging from 7 to 8 $R_{E}$. Note that the transit times for the two satellites through this region of the magnetosphere are the same within 3 min. During this period, apogee moved westward from noon to dawn, into midnight, and subsequently to the dusk sector of the equatorial magnetosphere. This interval is marked by several enhanced periods of magnetospheric activity, as measured by the 3 h Kp index shown in Figure 6a. Such periods are usually, but not always accompanied by enhanced solar wind dynamic pressures $P_{\text{SW}}$ depicted in Figure 6b. To better see gross correspondences, fiducial dotted lines are provided at select gross minima in $P_{\text{SW}}$. From close inspection, one can see that in many instances $P_{\text{SW}}$ and Kp enhancements appear coaligned. In other instances pressure enhancements occur at the leading edge or in the transition of Kp enhancements. Correlation analysis reveals a statistically significant moderate positive correlation between Kp and $P_{\text{SW}}$ with a correlation coefficient of r=0.43±0.02 to 99% confidence in the interval. To examine the ion response, Figure 6c gives the total ion density $N_{i}$ sampled within the magnetosphere at distances ranging from 7 to 8 $R_{E}$. Note, both inbound and outbound crossings of this region are included as indicated by the magnetic local time (MLT) values shown in Figure 6f (corresponding to a difference of ΔMLT∼4 h). The ion densities are shown to vary considerably, with strong enhancements (of a few to tens per cubic centimeter) occurring in association with gross enhancements in the Kp index and/or solar wind dynamic pressure $P_{\text{SW}}$. To examine the extent to which the ion density is correlated with $P_{\text{SW}}$, Figure 6g depicts the distribution of all ion measurements within 7–8 $R_{E}$ in the magnetosphere as a function of $P_{\text{SW}}$ and $N_{i}$. Though there is quite a bit of scatter in the data, the comparison indicates a positive correlation between $P_{\text{SW}}$ and $N_{i}$. Correlation analysis of data samples yielded a weak, but statistically significant, positive correlation with a coefficient of r=0.28±0.03 to 99% confidence. The trend is made more apparent by the log space bin averages and standard deviations of data samples indicated by the solid black dots and bars in Figure 6g. A regression analysis to the bin averaged data yielded the power law relation $N_{i}=10^{-0.36}P_{\text{SW}}^{0.42}$. Though not shown, the correlation is notably improved by considering only dayside measurements, which yielded a coefficient of r=0.36±0.03 to 99% confidence and a power law relation $N_{i}=10^{-0.39}P_{\text{SW}}^{0.61}$. The weak, but statistically significant, correlation indicates that other factors are at play that impact the correlation. Close inspection of separate inbound and outbound data time series on shorter time scales (not shown) suggests that a big contributing factor is that the ion density response in the magnetosphere is not homogeneous, with significant differences occurring in the inbound and outbound MLT transects of the 7–8 $R_{E}$ region. As will be explored and shown below, another contributing factor is that there exist different contributing sources to density that have different responses to $P_{\text{SW}}$. These correlated results indicate that significant enhancements to magnetospheric density occur during compressed and/or active magnetospheric conditions. During these periods, the cold to hot density ratios given in Figure 6d are observed to increase from nominal/quiet time levels of a few tenths to elevated levels ranging from above a factor of 2 and up to several tens in places. These elevated ratios are invariably associated with sudden drops in the cold ion temperatures to values ≲10 eV, as shown in Figure 6e, and thus drops in the cold to hot ion temperature ratio $T_{ic}/T_{ih}$ (not shown). The correspondence with temperature is further exemplified in Figure 6h, which shows the distribution of ion measurements as a function of $P_{\text{SW}}$ and the cold to hot ion temperature ratio $T_{ic}/T_{ih}$. This comparison reveals a negative correlation between $P_{\text{SW}}$ and $T_{ic}/T_{ih}$. Correlation analysis yielded a statistically significant weak negative correlation with coefficients of r=−0.23±0.03 for all measurements and r=−0.27±0.04 for dayside measurements only at 7–8 $R_{E}$ distances in the equatorial magnetosphere, respectively. Fits yielded the power relations $T_{ic}/T_{ih}=10^{-1.8}P_{\text{SW}}^{-0.41}$ for all measurements and $T_{ic}/T_{ih}=10^{-1.8}P_{\text{SW}}^{-0.32}$ for just dayside measurements. These trends in combination with the case examples in Section 3 demonstrate that the enhanced densities seen in Figure 6c are due to the appearance/intrusion of new cold dense low energy ions into the magnetosphere at these distances, as opposed to compression of existing plasma. Moreover, these correlative results make apparent the dramatic transition the magnetosphere undergoes from an initial quiet time state dominated by hot tenuous plasma sheet ions to a state dominated by the presence of cold dense low‐energy ions during enhanced magnetospheric activity and/or solar wind pressure. The duration of such cold dense plasma in the magnetosphere is multiscale in nature depending on the variability/duration of active conditions and/or solar wind pressure variations. On gross time scales enhanced cold plasma are shown to last anywhere from a few days to a significant fraction of a month (10–20 days), such as in May. Shorter time enhancements lasting from a few hours to a significant fraction of a day are also observed in response to short impulsive changes in the solar wind dynamic pressure and/or Kp.

**Figure 7:**
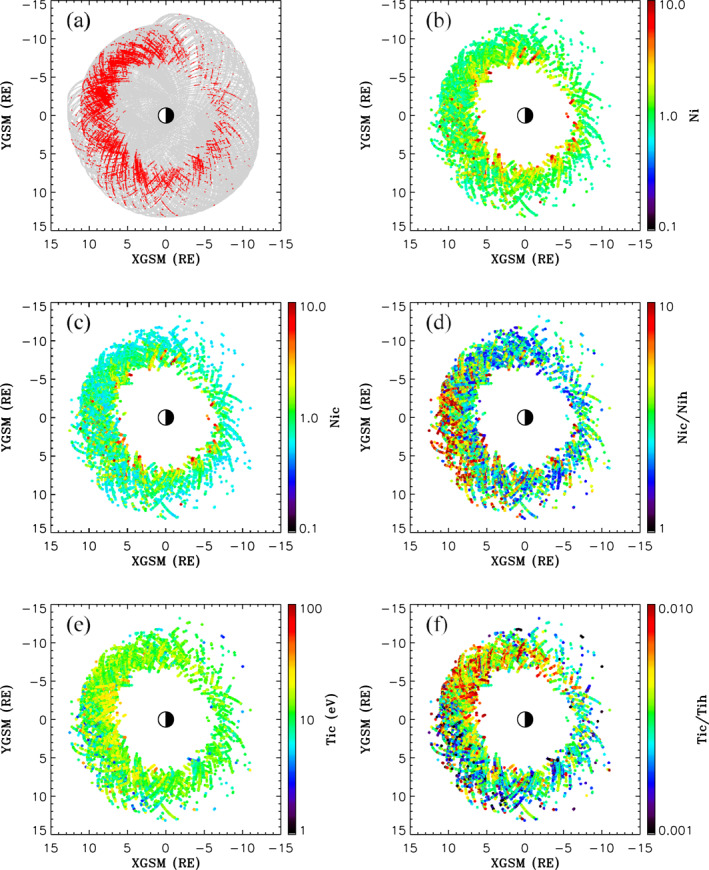
Shows (a) Plasma distribution samples from Time History of Events and Macroscale Interactions during Substorms A and D spanning June 21, 2016 to August 31, 2017 with cold dense ion samples indicated in red, (b) Total ion density, (c) Cold ion density, (d) Cold to hot ion density ratio, (e) Cold ion temperature, and (f) Cold to hot ion temperature ratios.

To examine the distribution of cold dense ions in the outer magnetosphere, Figure 7 shows ion densities and temperatures sampled by the THEMIS A and D spacecraft in the interval spanning June 21, 2016 to August 31, 2017 at geocentric distances >$6\;R_{E}$. Note, local noon is toward the left, midnight is toward the right, dawn toward the top, and dusk toward the bottom of each plot. All 3s resolution ion measurements as a function of X‐GSM and Y‐GSM sampled during this time are shown in Figure 7a with identified cold dense ions at >$6R_{E}$ distances in the magnetosphere indicated by the red dots. These events were selected based on cold densities $N_{ic}\geq 0.5\;{cm}^{-3}$ and ratios between cold and hot ion densities $N_{ic}/N_{ih}\geq 1.5$. With these selection criteria, the choice of the 150 eV threshold to separate hot and cold ion populations is quite good for isolating the targeted dense low‐energy ions reported in this study. These cold ions are predominantly observed to have characteristic energies (energy flux divided by the number flux) and bulk energies (perpendicular, parallel, or antiparallel to the background magnetic field) at several tens of eV and below. Particularly, 99.7% of the events in Figure 7 have characteristic energies in the range from 3 to 62 eV with a peak value of 14 eV (not shown).

It is apparent in Figure 7a, that cold dense plasma is a persistent feature of the dayside magnetosphere in the equatorial plane, with events encountered on most orbital passes from dawn to dusk. Dense low energy ions are observed in the nightside sector (X‐GSM <0), however the number of events is substantially reduced. The total ion and cold ion densities are respectively shown in Figures 7b and 7c. The low energy ions have densities that are typically $∼\;1-2\;{cm}^{-3}$, with some examples reaching up to a few tens per cubic centimeters. With some exceptions in the postnoon sector of the dayside magnetosphere, there is a tendency for low energy ion densities and hence total ion densities to be higher closer to Earth (yellow to red signatures in Figures 7b and 7c). As suggested in Figure 6, this may be due to these ion contributions being observed during compressed magnetospheric conditions associated with enhanced solar wind dynamic pressure. The cold to hot ion density ratios $N_{ic}/N_{ih}$ shown in Figure 7d have values ranging from our imposed lower bound limit of 1.5 and up to 100, with a mean (median) value of 4.4 (3.2). Values for $N_{ic}/N_{ih}$ are particularly enhanced (red) from prenoon to near dusk. These are likely attributed to relatively cold plasmaspheric plume ions. This assertion will be examined in more detail below.

Figure 7e shows temperatures of the low energy ions in our database as a function of X‐GSM and Y‐GSM. The dense low energy ions are relatively cold with temperatures ranging from 1.4 to 40 eV, with a mean (median) value of 13 eV (12 eV). There is a tendency for the low energy ions to be relatively colder (blue) in the postnoon sector at larger radial distances and a little warmer (yellow) at closer distances about noon, likely indicative of distinct ion populations, which will be examined in more detail below. The cold to hot ion temperature ratios $T_{ic}/T_{ih}$ are shown in Figure 7f. The ratios range from $T_{ic}/T_{ih}∼3\times 10^{-4}-∼0.02$, with a mean (median) of $4.1\times 10^{-3}$ ($3.5\times 10^{-3}$). Also, the ratios $T_{ic}/T_{ih}$ show a dawn‐dusk asymmetry, attributed to the preferential occurrence of relatively colder ions on the dusk side of the magnetosphere (Y‐GSM >0).

**Figure 8:**
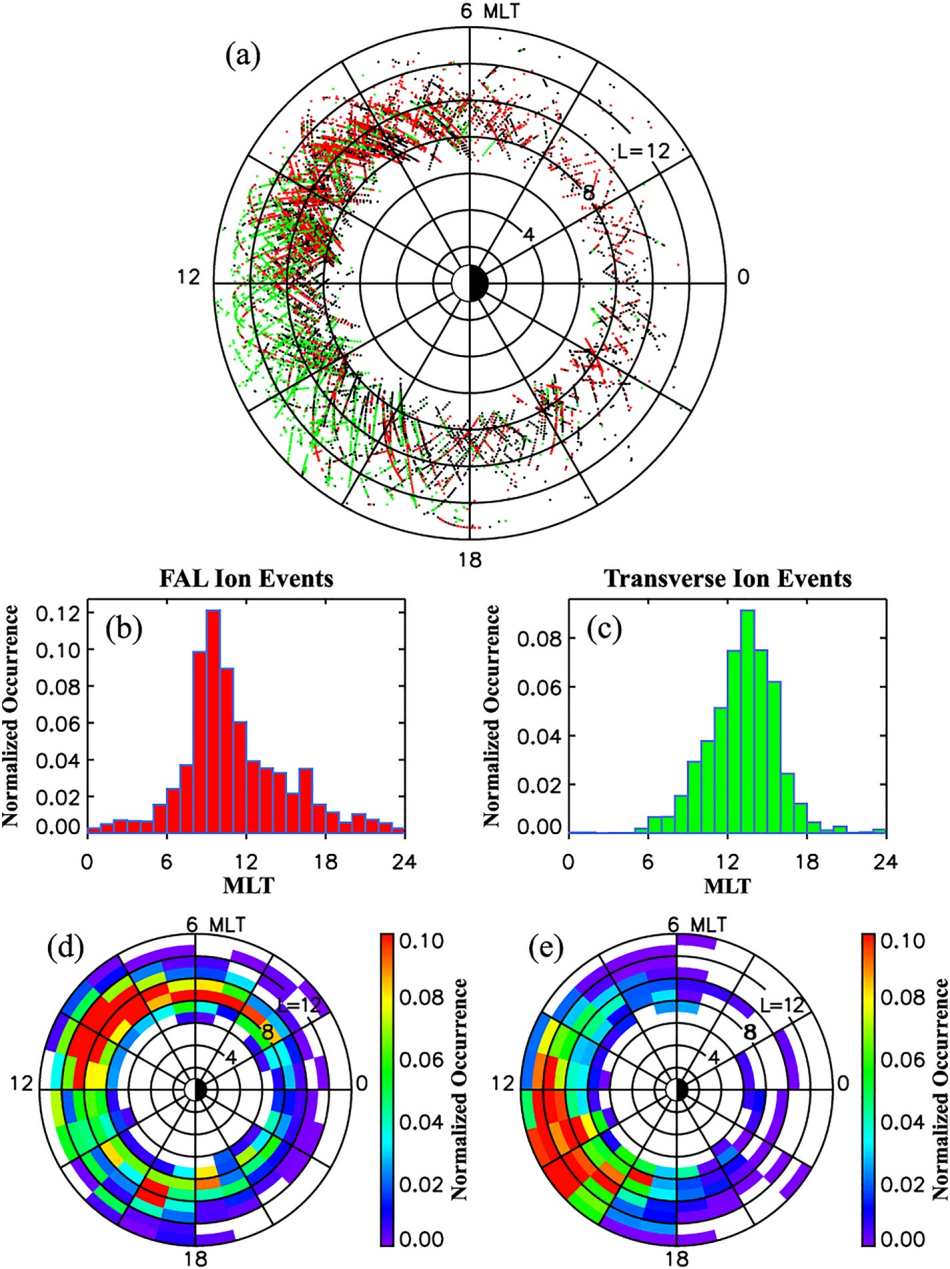
(a) Distribution of cold ion events as a function of L‐shell and magnetic local time (MLT) (with noon MLT to the left) with green indicating transverse ions, red field aligned ions (uni‐ or bi‐directional) and black all others. Also shown are histograms of MLT occurrences for (b) Field‐aligned ions, and (c) Transverse ions, and histograms of L‐shell versus MLT occurrences for (d) Field‐aligned ions, and (e) Transverse ions.

The case studies in Section 3 demonstrated that the cold dense ion distributions in the magnetosphere have differing flux/flow characteristics, suggestive of different contributing source populations. Particularly, the cold ions are typically observed to have either preferential perpendicular fluxes, field‐aligned fluxes, or a combination of both. Ions with preferential perpendicular fluxes are observed to be associated with strong convection (namely with convected drift energy being much greater than both the ion thermal energy or parallel kinetic energy). Ions with preferential field‐aligned fluxes show up as either unidirectional or counterstreaming beams with parallel kinetic energies that are much larger than the convection energy. To examine how these classes vary as a function of geophysical location, Figure 8a shows a plot of all cold dense ion samples from THEMIS A and D for the interval from June 21, 2016 to August 31, 2017 as a function of L‐shell and MLT (with noon MLT to the left). The events are color coded according to their flux characteristics, with green indicating ions with preferential perpendicular/transverse fluxes, red parallel flux (either uni‐ or bi‐directional), and black all others. Here, preferential perpendicular flux ion events are defined as those having both peak parallel to perpendicular flux ratios $J_{E∥}/J_{E⊥}<0.5$ and peak antiparallel to perpendicular flux ratios $J_{E-∥}/J_{E⊥}<0.5$ at energies <150 eV. In contrast preferential parallel flux ion events are defined as those having the peak parallel to perpendicular flux ratios $J_{E∥}/J_{E⊥}>2$ and/or peak antiparallel to perpendicular flux ratios $J_{E-∥}/J_{E⊥}>2$ at energies <150 eV. All other events correspond to those low‐energy dense ion events that do not meet these criteria and include weakly parallel and perpendicular anisotropy events in addition to isotropic events. As demonstrated in Figure 8a, the great majority of events fall within the preferential perpendicular or parallel class of events. These two classes show the characteristic pattern of previously reported plume (e.g., Chappell, 1974; Goldstein, 2006) and cloak/bidirectional ions (e.g., Chappell et al., 1987, 2008) in the magnetosphere. Here, the convection dominated/preferential transverse flux events correspond to plume ions, while the field‐aligned (uni‐directional or bi‐directional) flux events include cloak and other ion outflow types of populations. In this study, we will focus on and refer to these two classes as transverse and field‐aligned (FAL) ion events. Figure 8a shows that both classes of ions often span a large spatial extent of an orbital transect (ΔL∼ 1–5). Moreover, the ions are observed to occur over multiple sequential orbits, indicating that such populations are continuously being resupplied/replenished to the magnetosphere. It is also important to note that the dense cold plasma populations along a transect are not spatially uniform but are often intermittent indicating that these ions result in spatial inhomogeneities in the magnetosphere density.

The MLT occurrences of FAL and transverse ion events are given in Figures 8b and , respectively. The occurrence rate at a given MLT bin has been normalized to account for sampling biases and is defined as the number of 3s resolution dense cold ion measurements of a given type (FAL or transverse) in the magnetosphere at geocentric distances >$6R_{E}$ divided by the total number of 3s resolution measurements in that bin from fast and slow survey modes. This is complemented with occurrences versus L‐shell and MLT presented in Figures 8d and 8e for FAL and transverse ions, respectively. The occurrence at a given L—MLT bin is defined as the number of dense cold ion measurements of a given type (FAL or transverse) divided by the total number of measurements in that bin. The results show that FAL ion events are seen at all MLTs but predominantly occur between dawn and noon with peak occurrence at ∼0930 MLT. While events occur further out, FAL ion peak occurrences tend to occur at L values from 8 to 12 (red in Figure 8d), with further (closer) distance ranges occurring in the prenoon (dawn) sector. There is also evidence of enhanced occurrences near dusk. Though there were differences in how the data was selected, binned, and how occurrences were normalized, the occurrence distribution of FAL ions shown in Figure 8 is qualitatively similar to the distribution of occurrences of bidirectional ions reported in other studies (Chappell et al., 2008; Nagai et al., 1983). Namely, all show a strong preferential dawnside occurrence bias with a notable dusk population. In our case the dusk ion occurrence is most enhanced pre‐dusk at L ∼ 9–10, which is more consistent with those reported in the study by Nagai et al. (1983), while those in Chappell et al. (2008) were post‐dusk. The differences may be due to differing conditions that characterize each of the different event databases, such as IMF orientations. The study by Chappell et al. (2008) suggested, via trajectory mappings of protons under southward IMF conditions, these correspond to polar wind ions that merely bounce between hemispheres, before escaping to the magnetosheath. This was explained to be the result of orbits being near the separatrix, where corotational and convection electric fields are in opposite directions and cancel. In our case, the dusk population is exemplified by the 08 May 2017 case study event shown in Figure 5 above, which occurred under northward IMF conditions. How such mappings will change for these conditions are unclear. Perhaps something similar happens but the source location and potentially the source type may change. Also, it should be noted that the dawnside bias in these low‐latitude/equatorial occurrences shows remarkable similarity to that found in the flux distribution and occurrences of dayside cleft associated upwelling ions observed in high‐latitude measurements from low‐altitude polar orbiting spacecraft reported in the literature (e.g., Lockwood et al., 1985; Redmon et al., 2012), suggestive of a possible significant contributing source.

In contrast, transverse ions tend to occur over the prenoon to dusk sectors of the magnetosphere with peak occurrence at ∼1330 MLT (see Figure 8c). Moreover, peak occurrences are found to occur at larger distances from Earth at L values from ∼8 to 14 (red in Figure 8e). The occurrence distribution in L‐MLT space is complementary and qualitatively similar to those reported in the study by Chen and Moore (2006), which was based on 3.5 years of Polar‐TIDE data sampled in the dayside magnetosphere at magnetic latitudes of ±60° and at distances closer to Earth (≲$9R_{E}$) than presented here. Particularly noteworthy is that the occurrences in both studies show increases at larger distances that are closer to the magnetopause. This may be related to the effects of magnetopause motion and/or enhanced reconnection flows, which enable the ions to occur at energies above the spacecraft floating potential energy barrier and be measured by the ESA detectors (McFadden et al., 2008b; Sauvaud et al., 2001).

### 4.1 Occurrence Dependencies on *Kp*

**Figure 9:**
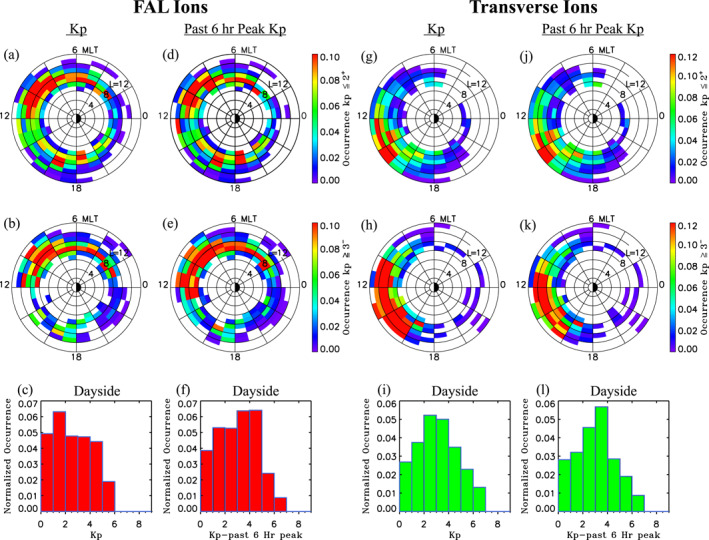
Normalized occurrences as a function of L and magnetic local time (MLT) for (a, g) $Kp\leq 2^{+}$ and (b, h) $Kp\geq 3^{-}$, and as a function of (c, i) Kp for field‐aligned (FAL) ions (a)–(c) and transverse ions (g)–(i). Also shown are normalized occurrences as a function of L and MLT for (d, j) past 6 h peak $Kp^{6h}\leq 2^{+}$ and (e, k) $Kp^{6h}\geq 3^{-}$, and as a function of (f, l) $Kp^{6h}$ for FAL ions (d)–(f) and transverse ions (j–l).

To examine connections with magnetospheric activity, Figure 9 shows the dependence of occurrences on Kp for the two classes of ions at geocentric distances >$6R_{E}$. Figures 9a and 9b present normalized occurrences of FAL ions as a function of L and MLT for values of $Kp\leq 2^{+}$ and $Kp\geq 3^{-}$, respectively. The normalized occurrences of FAL ions in the dayside as a function of Kp are also given in Figure 9c. The results show that cold dense FAL ions are observed to occur over a broad range of Kp values, but have a tendency to occur under relatively quiet conditions, with peak Kp∼1−2 in Figure 9c. The occurrence distribution of FAL ions in L—MLT space during quiet conditions given in Figure 9a shows that these ions are more broadly distributed, being seen at virtually all MLTs, with the most pronounced occurrences seen from morning to noon at L∼ 7–12. Additional localized peaks in occurrences are also seen near dusk. In contrast Figure 9b shows that the FAL ions during more active periods are more concentrated in the morning to noon sector of the magnetosphere with somewhat depressed occurrences seen at most of the other regions. It is worth mentioning that the tendency for the FAL ions to be observed during quieter magnetospheric conditions in the pre‐noon sector is also similar to the observed upwelling ion trends reported in past studies (Redmon et al., 2012). The similar behavior supports the notion that the upwelling ions observed in the vicinity of the cusp/cleft region at low altitudes are the source of the FAL ions observed by THEMIS in the equatorial magnetosphere. According to the study by Redmon et al. (2012), the dawnward bias is the result of the peak of upwelling low energy ions occurring near dawn combined with the effects of the peak magnetospheric energy accelerating the ions in the cusp near noon.

To examine connections with previous magnetospheric activity, Figure 9f shows normalized occurrences of dayside FAL ions as a function of 6‐h peak values of $Kp^{6h}$. Figure 9f shows a distinct increase in the FAL ion occurrences during more moderate to active magnetospheric conditions that transpire within the previous 6 h, with occurrences peaking at $Kp^{6h}∼3-5$. To examine quiet versus active differences in their global distribution, Figures 9d and 9e present normalized occurrences of FAL ions as a function of L and MLT for 6‐h peak values of $Kp^{6h}\leq 2^{+}$ and $Kp^{6h}\geq 3^{-}$, respectively. Similar to its quiet time counterpart with $Kp\leq 2^{+}$ shown in Figure 9a, the quiet time distribution with $Kp^{6h}\leq 2^{+}$ in Figure 9d shows pronounced occurrences from morning to noon at L spanning from 7 to 12, with additional localized occurrence peaks seen near dusk. However, the active time distribution with $Kp^{6h}\geq 3^{-}$ in Figure 9e is notably enhanced at all L—MLT regions relative to the distribution given Figure 9b. In comparison to the distribution with $Kp^{6h}\leq 2^{+}$ in Figure 9d, Figure 9e shows that the FAL ion occurrences are notably higher in the dawnside magnetosphere (red from 0200 to 1200 MLT at L∼8−11) for events associated with moderate to active magnetospheric conditions that transpire within the previous 6 h ($Kp^{6h}\geq 3^{-}$). The FAL ion occurrences in Figure 9e are also seen to be notably depressed in the duskside magnetosphere. The combined results presented in Figures 9a–9f demonstrate that the FAL ions in the dawnside become more prominent in the magnetosphere during the relatively quieter periods that follow more enhanced activity, such as may occur during the recovery phase of geomagnetic storms. In contrast they suggest that the FAL events near dusk are associated with sustained quiet time conditions.

Figures 9g and 9h present normalized occurrences of transverse ions as a function of L and MLT for values of $Kp\leq 2^{+}$ and $Kp\geq 3^{-}$, respectively. Also shown in Figure 9i is normalized occurrences of transverse ions as a function of Kp. In contrast to FAL ions, these results indicate that the transverse ions are preferentially observed during moderately enhanced magnetospheric conditions with peak occurrences at values of Kp∼2−4 (see Figure 9i). The normalized occurrence distribution as a function of L and MLT during quiet magnetospheric conditions ($Kp\leq 2^{+}$) in Figure 9g reveals that the transverse ions preferentially occur from the prenoon sector to postdusk with peak occurrences limited to the postnoon sector. In contrast Figure 9h reveals that the transverse ions during more active magnetospheric conditions preferentially occur in the dayside region of the magnetosphere, with notably enhanced occurrences seen over the broad range of MLTs from 10 to 16 h and L values from 10 to 12. This result highlights the strong impact of enhanced convection in draining cold dense plasmaspheric material to the dayside region of the magnetosphere.

The relation of transverse ion occurrences to past 6 h activity conditions was also examined. Figure 9l, which shows occurrences of transverse ions in the dayside as a function of $Kp^{6h}$, reveals the peak occurrence has slightly shifted to higher values of $Kp^{6h}∼3-4$. The occurrence distribution as a function of L and MLT for the two different ranges of $Kp^{6h}$ depicted in Figures 9j and 9k still show similar behavior as seen for global occurrences versus Kp shown in Figures 9g and 9h. Namely, the transverse ion occurrences are strongly enhanced over a broader range of MLTs (9–16 h MLT) and L∼10−12 during more active magnetospheric conditions. These comparisons indicate that the transverse ions in the dayside region are the result of sustained (∼6h or less) moderate to very active magnetospheric conditions.

### 4.2 Occurrence and Density Dependencies on Solar Wind Pressure

**Figure 10:**
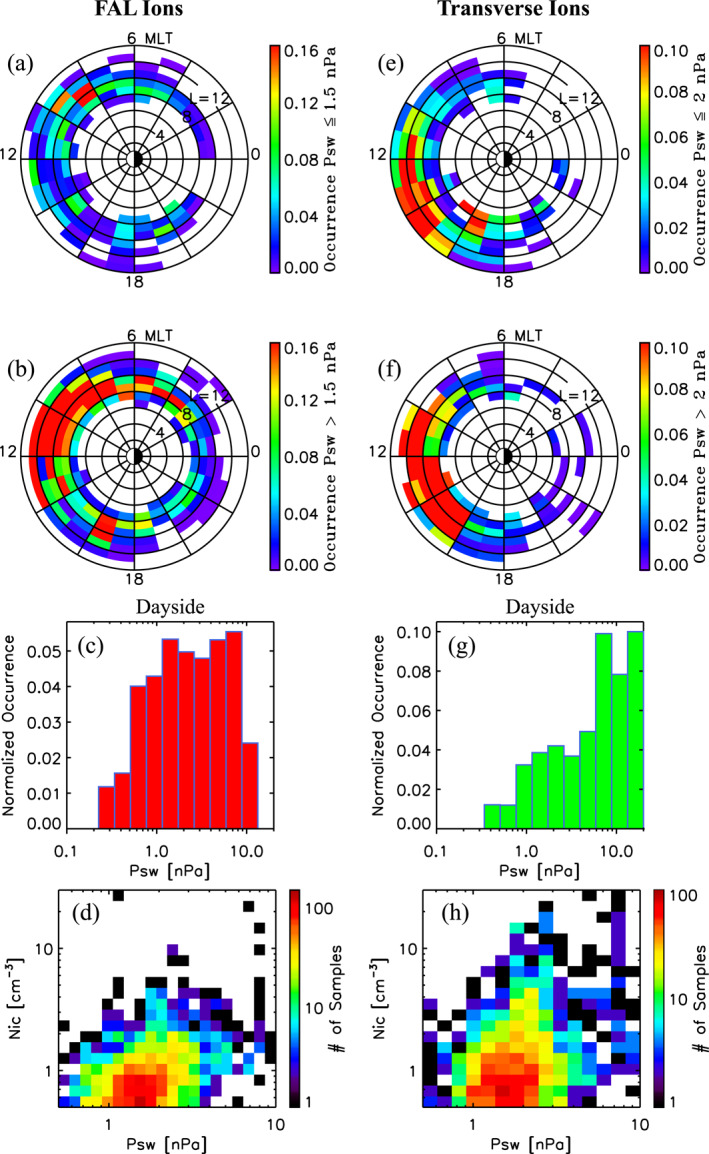
Field‐aligned (FAL) ion normalized occurrences as a function of L and magnetic local time (MLT) for solar wind pressures (a) $P_{\text{SW}}\leq 1.5$ nPa and (b) $P_{\text{SW}}>1.5$ nPa, (c) Dayside FAL ion normalized occurrences as a function of $P_{\text{SW}}$, and (d) Distribution of dayside FAL ion events versus $P_{\text{SW}}$ and density. Transverse ion normalized occurrences as a function of L and MLT for (e) $P_{\text{SW}}\leq 2$ nPa and (f) $P_{\text{SW}}>2$ nPa, (g) Dayside transverse ion normalized occurrences as a function of $P_{\text{SW}}$, and (h) Distribution of dayside transverse ion events versus $P_{\text{SW}}$ and density.

Figure 6 discussed above suggests that cold ions in the outer magnetosphere are controlled by solar wind dynamic pressure $P_{\text{SW}}$. To examine how $P_{\text{SW}}$ affects the occurrence of FAL ions at geocentric distances >$6R_{E}$, Figures 10a and 10b depict normalized occurrences in L and MLT for $P_{\text{SW}}\leq 1.5$ nPa and $P_{\text{SW}}>1.5$ nPa, respectively. The occurrences of dayside FAL ion events as a function of $P_{\text{SW}}$ are also given in Figure 10c. The results show that dayside FAL ion event occurrences increase sharply till about 1.2 nPa, after which they plateau (or increase slightly) at higher pressure values (see Figure 10c). Examination of the occurrence distributions in L—MLT depicted in Figures 10a and 10b reveal that solar wind pressures above 1.5 nPa are associated with significantly higher occurrences over all MLTs, with the most pronounced effect seen prior to local noon over the broad L‐shell range from 8 to 13. A distinct enhancement is also seen in the dusk sector in Figure 10b.

The occurrences of transverse ions are also observed to be strongly affected by the solar wind pressure. This is revealed in Figure 10g, which shows a strong increase in the dayside transverse ion occurrences with increasing solar wind dynamic pressure. Unlike the FAL ions, which showed a plateauing effect, the occurrences of transverse ions, more or less, continuously increase with increasing pressure. To examine the global response, Figures 10a and 10b depict normalized occurrences of transverse ions in L and MLT for $P_{\text{SW}}\leq 2$ nPa and $P_{\text{SW}}>2$ nPa, respectively. These results demonstrate that higher solar wind dynamic pressures are associated with a significant increase in the occurrences of transverse ions in the dayside magnetosphere from 0900 to 1600 MLT over a large L extent from 8 to 12. As one may expect from compressed conditions associated with enhanced solar wind pressure, the larger occurrences (red in Figure 10f) are seen closer to Earth than those that occur during $P_{\text{SW}}\leq 2$ nPa, which are seen to extend from L values from 10 to 14 in the postnoon sector of Figure 10e.

To examine relationships between pressure and density, Figures 10d and 10h show the distribution of dayside low energy FAL ion and transverse ion densities as a function of solar wind pressure $P_{\text{SW}}$, respectively. The comparisons indicate that both dayside FAL and transverse low energy ion densities are correlated with solar wind dynamic pressure. Correlation analysis of pressure and density measurements yielded weak positive, though significant, correlations with coefficients of 0.34±0.04 and 0.32±0.05 to 99% confidence found for dayside FAL and transverse ion events, respectively.

These combined results demonstrate that solar wind dynamic pressure is an important controlling factor in both the amount of these two classes of low energy ions occurring in the magnetosphere and their densities. The weak positive correlations indicate that other factors are also at play, which we leave for future work.

How enhanced solar wind dynamic pressure is affecting ion outflow from the ionosphere that ultimately determines the FAL ion occurrences and density in the equatorial magnetosphere is not fully understood. This is owing to the fact that solar wind pressure enhancements generally coincide with other factors, such as the magnitude of the solar wind electric field, and the magnitude and sign of the IMF $B_{z}$. Compression of the magnetosphere closer to Earth due to solar wind dynamic pressure enhancements can result in density increases of the FAL ions observed therein. However, the fact that the cold ion density increases are accompanied by drops in temperature as shown in Figure 6 suggests that the correlation between FAL ion density and solar wind pressure is more than just simple compression of pre‐existing FAL ions, but that solar wind dynamic pressure is impacting the supply of source ion outflow. Observations suggesting that solar wind dynamic pressure is playing a role in ion outflow have been reported (e.g., Cully et al., 2003; Fuselier et al., 2001; Lennartsson et al., 2004; Moore et al., 1999). Particularly, Cully et al. (2003) reported statistically significant correlations between solar wind dynamic pressure and the fluences of low energy (<1–70 eV) ion outflow observed at low‐altitudes in the high‐latitude polar regions by Akebono.

Solar wind dynamic pressure may be impacting ion outflow by enhancing “soft” (≲1 keV) electron precipitation and/or electromagnetic energy flow (DC Poynting flux) into the ionosphere, both of which are important ion outflow controlling factors (e.g., Strangeway et al., 2005; Zhao et al., 2020). In support of the former, the simulation results of Damiano et al. (2010) showed that increases in $P_{\text{SW}}$ lead to increased cusp electron precipitation, which effectively enhances the available supply of upflowing ions for outflow. Dang et al. (2018) reported simulation results that implicate the solar wind pressure as the most important controlling factor of both the hemispheric precipitation rate and the hemispheric power of precipitating soft electrons in the cusp proper. A strong correlation between densities in the cusp and solar wind density was reported by Walsh et al. (2016), which presents indirect observational evidence of a connection with $P_{\text{SW}}$. Given that solar wind densities are generally associated with enhancements in $P_{\text{SW}}$, the implication of this result is that the density of the soft electron precipitation increases with enhancements in $P_{\text{SW}}$. Zhou et al. (2003) reported enhanced soft electron precipitation in the auroral zone in association with sudden increases in solar wind dynamic pressure. Thus, it seems plausible that the observed correlations between ion outflow flux and soft electron precipitation densities and fluxes reported (Strangeway et al., 2005; Zhao et al., 2020) could arise from enhanced solar wind dynamic pressure, but this connection needs further study.

Enhanced electromagnetic energy flows (Poynting flux) into the ionosphere in association with solar wind dynamic pressure have also been reported (e.g., Li et al., 2011; Shi et al., 2017). In the study by Li et al. (2011) large DC Poynting fluxes associated with enhanced convective flow channels in the dayside ionosphere were observed under northward IMF with a large IMF $B_{y}$ component under enhanced solar wind dynamic pressures. This is particularly relevant in our case here in that, as will be shown below, the FAL ions are associated with northward IMF conditions. The enhanced Poynting fluxes were associated with intensified ionospheric Joule heating, which can lead to enhanced ion outflow rates. Positive correlations between solar wind dynamic pressure and field‐aligned currents (e.g., Korth et al., 2011; Wing et al., 2007), and associations with convective flows/electric fields (e.g., Boudouridis et al., 2007; Li et al., 2011), which are relevant to Joule heating, have also been reported. How these correspondences with pressure relate to ion outflow have not been firmly established and will require further study.

The manner in which enhanced solar wind dynamic pressure is impacting the density and occurrences of the transverse ions is also not fully understood. The peak transverse ion occurrences in the post‐noon sector indicate that these are due to plasmaspheric plume ions. A feasible explanation for the correlation between $P_{\text{SW}}$ and the cold transverse ion density is that it is due to the compression of the magnetosphere closer to Earth by enhanced solar wind dynamic pressure, the effect of which increases the pre‐existing plume and/or the source plasmaspheric ion densities. The dominant factor in the occurrence of plume ions in the outer magnetosphere is well known to be enhanced dayside magnetic reconnection driven convection, which effectively expels these ions from their initial location in the dusk plasmasphere toward the magnetopause (e.g., Grebowsky, 1970; Chen & Wolf, 1972; Goldstein & Sandel, 2005). Thus, the enhanced occurrences could be an indication that enhancements in the solar wind dynamic pressure are enhancing global magnetospheric convection.

Magnetospheric convection is controlled by dayside and nightside reconnection. Observational studies signifying that sudden increases in solar wind dynamic pressures are enhancing dayside and/or nightside reconnection rates, hence global magnetospheric convection, have been reported (e.g., Boudouridis et al., 2007, 2008, 2021). The simulations performed by Connor et al. (2014) also showed that strong pressure enhancements can intensify dayside and nightside reconnection during southward IMF conditions. These combined results show that pressure can indeed affect magnetospheric convection. However, the extent to which pressure affects the convection process and subsequent expulsion of plasmaspheric ions to form plumes and their consequential effects require further study in observations and simulations.

### 4.3 Dependencies on IMF Orientation

**Figure 11:**
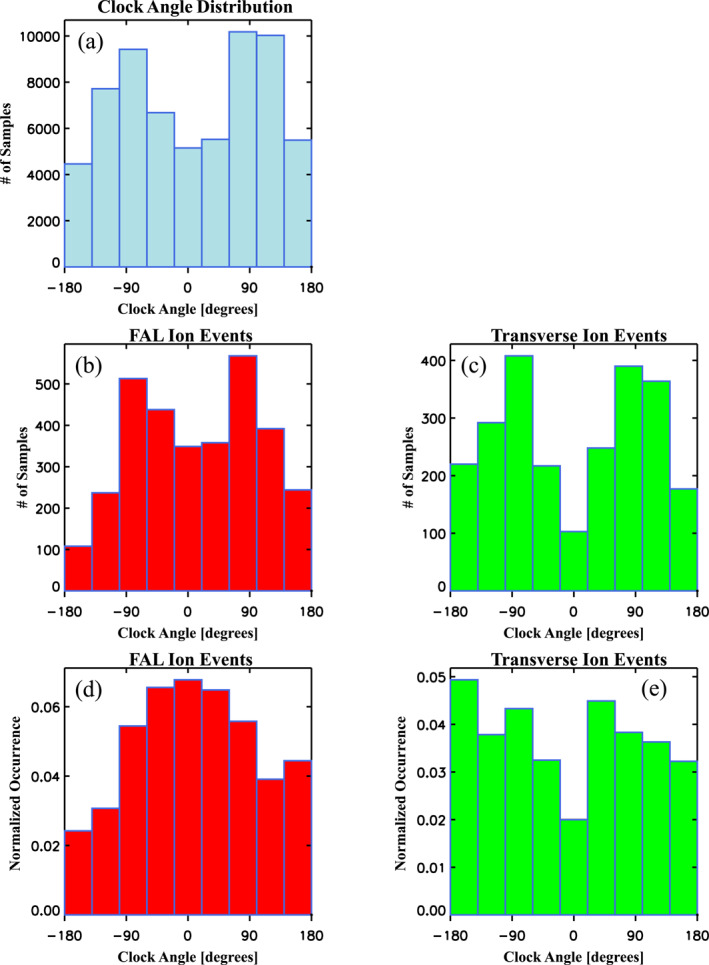
Histograms of the IMF magnetic field clock angle for (a) All dayside magnetospheric measurements, (b) Dayside field‐aligned (FAL) ion events, and (c) Dayside transverse ion events, and normalized occurrences of (d) Dayside FAL and (e) dayside transverse ion events as a function of clock angle.

The occurrences of dayside FAL and transverse ion events are sensitive to the IMF orientation. To demonstrate this, Figure 11 depicts histograms and normalized occurrences of the IMF clock angle defined as $\theta =\arctan\left(B_{\text{GSMy}}/B_{\text{GSMz}}\right)$, which is the orientation of the magnetic field in the GSM Y−Z plane. In this plane 0° corresponds to the +Z‐GSM or northward direction, ±180° to −Z‐GSM or southward direction, +90° to +Y‐GSM or eastward/duskward direction, and −90° to −Y‐GSM or westward/dawnward direction. To estimate θ, we used the 5 min resolution IMF magnetic field data shifted in time to the bow shock nose, which were interpolated to the time tags for cold dense ions measured by the THEMIS A and D spacecraft in the dayside magnetosphere. The distribution of IMF clock angles for all dayside magnetospheric measurements given in Figure 11a is peaked at ±90°, owing to the statistical expectation for the IMF to be at the Parker spiral at quiet times (i.e., oriented within the ecliptic plane at ±45° from the Earth‐sun line). Similarly, the clock angle distribution of dayside FAL ion and transverse ion events are respectively shown in Figures 11b and 11c, which also reveal peaks at ±90° owing to the typical solar wind IMF orientation. The normalized occurrences for dayside FAL and transverse ions are shown in Figures 11d and 11e, respectively. In order to remove the IMF orientation and sampling rate biases these were determined as the clock angle distribution of a given class of ion (FAL or transverse) divided by the clock angle distribution of all dayside magnetospheric measurements. The normalized occurrence distribution for dayside FAL ions shown in Figure 11d shows a broad peak between ±90°, corresponding to preferential northward IMF $B_{z}$ orientations. In contrast the occurrence rate for dayside transverse ions shown in Figure 11e is peaked at −180° corresponding to southward IMF, with a minimum (by a factor of 2 or more) for θ = 0° or northward oriented IMF.

**Figure 12:**
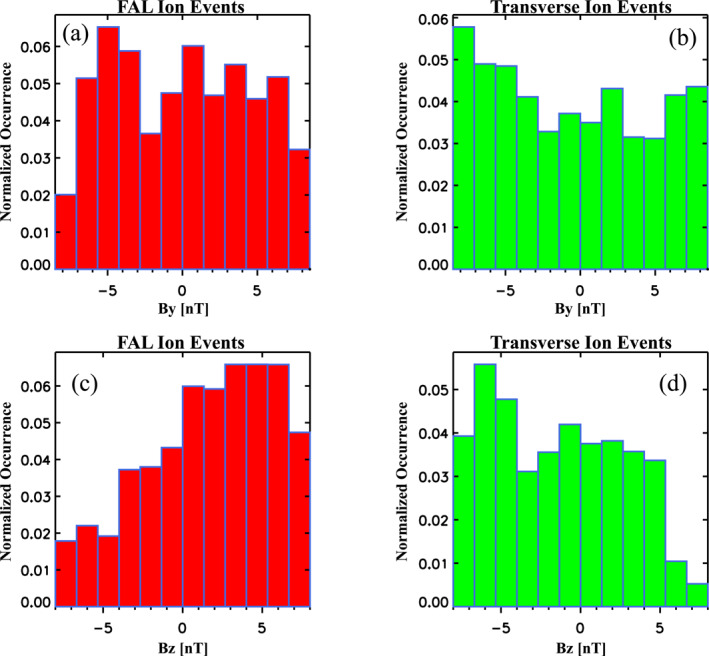
Normalized occurrences of (a) Field‐aligned (FAL) and (b) Transverse ion events in the dayside as a function of IMF $B_{y}$, and normalized occurrences of (c) FAL and (d) Transverse ion events in the dayside as a function of IMF $B_{z}$.

Figure 12 shows the complementary dependence of normalized occurrences for the two classes of dayside cold ion events with IMF $B_{y}$ and $B_{z}$. Consistent with the clock angle results above, occurrences of FAL ions show a clear dependence on IMF $B_{z}$, with maximum values peaking at larger positive (northward) IMF $B_{z}$ orientation (see Figure 12c). However, no clear correspondence between FAL ion occurrences and IMF $B_{y}$ is seen in Figure 12a. In contrast the occurrences for the transverse ions show a peak at larger negative (southward) IMF $B_{z}$ orientations in Figure 12d. In addition Figure 12b indicates IMF $B_{y}$ also appears to impact transverse ion occurrences, with a slight preference toward larger negative $B_{y}$ values. This result is consistent with those reported by Chen and Moore (2006), who showed a similar dependence with transverse moving thermal ions at closer distances.

These results in combination with the clock‐angle dependencies described above suggest that the IMF orientation and size are important controlling factors that distinguish between the ions with differing pitch‐angle characteristics (transverse vs. uni‐ or bi‐directional) in the dayside magnetosphere. Also, these dependencies indicate that the two classes of cold dense ions are associated with dramatically differing reconnection driven/global magnetospheric convection conditions. On the one hand, given their clear association with northward IMF conditions, the FAL ions (e.g., which are predominantly cloak ions) in the dayside equatorial magnetosphere favor the solar wind‐magnetospheric coupling conditions that arise from lobe reconnection that occurs tailward of the cusp. Such an association is contrary to the generally accepted paradigm and particle tracing model assumptions used to determine the source of these ions, which presume steady southward IMF conditions (e.g., Chappell et al., 2008). This coupled with the fact that the FAL ions are associated with quiet magnetospheric conditions following more active periods, places important additional constraints that need to be taken into consideration in further modeling of the source properties of these ions.

On the other hand, the transverse ions, being associated with enhanced IMF $B_{z}$ southward conditions, are consistent with the current body of observational evidence that demonstrates these are the result of enhanced sunward magnetospheric convection at low latitudes driven by reconnection at the dayside magnetopause (e.g., Goldstein, 2006, and references therein). Our results in combination with those of Chen and Moore (2006), also indicate the importance of IMF $B_{y}$ effects in the occurrences/distribution of transverse ions in the dayside magnetosphere that extend out to the magnetopause.

### 4.4 Density, Temperature, and Flow Properties of Transverse and FAL Ions

**Figure 13:**
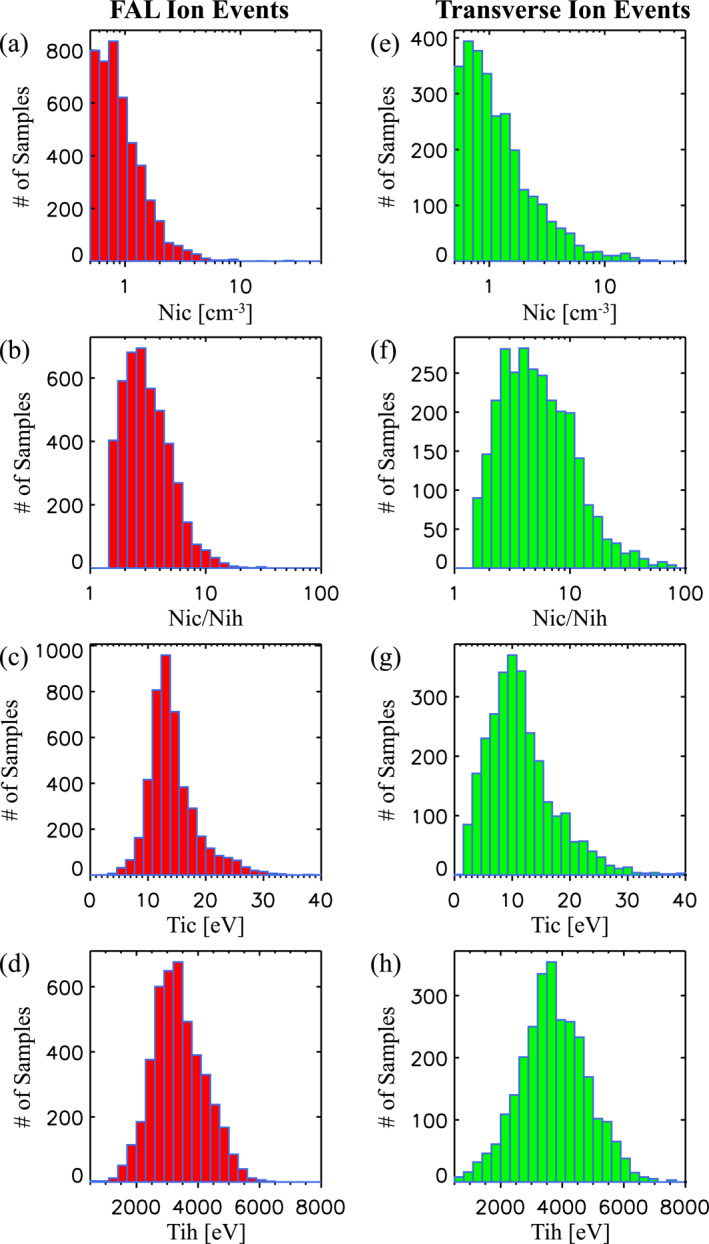
Histograms of (a and e) Cold ion densities, (b and f) Cold to hot density ratios, (c and g) Cold ion temperatures, and (d and h) Hot plasma sheet temperatures associated with field‐aligned ions (a–d) and transverse ions (e–h), respectively.

Figure 13a shows the density distribution of the low energy FAL ions at geocentric distances >$6R_{E}$. The FAL ion densities span from 0.5 to ∼10 ${cm}^{-3}$, with a mean (median) of ∼1 ${cm}^{-3}$ (0.8 ${cm}^{-3}$). Figure 13b shows that this population is relatively dense compared to the hotter plasma sheet population, yielding cold to hot density ratios that range from 1.5 to 20, with a typical value of $3.1_{-1.2}^{+1.9}$ and median of 2.9. As a comparison, Figure 13e reveals that the transverse ions span a broader range of densities with values ranging from 0.5 to 20 ${cm}^{-3}$, with a mean (median) of 1.7 ${cm}^{-3}$ (1 ${cm}^{-3}$). These densities are not resolvable in global plasmaspheric images, which have unambiguously verified the structure and dynamics of plumes above 40 ${cm}^{-3}$ (e.g., Goldstein, 2006). To get an idea of what these transverse ions densities represent at a canonical source location at L=4, we computed an approximate density using the relation $N\propto L^{-4}$ to account for flux expansion (Chappell, 1974). Based on this relation, the densities observed correspond to mapped values at L=4 ranging from tens to hundreds of ${cm}^{-3}$, with typical values of $60_{-30}^{+70}\;{cm}^{-3}$. As shown in Figure 13f, the locally measured transverse ion density values are associated with much larger cold to hot density ratios that range from 1.5 to ∼ 100 with a typical value of $5.2_{-2.7}^{+5.6}$ and median of 4.8.

Figures 13c and 13g display the temperature distribution of FAL and transverse ion events, respectively, at geocentric distances >$6R_{E}$. The FAL ions have temperatures that are typically 14±4 eV with a median of 13 eV. The transverse ions have temperatures that are typically 11±6 eV with a median of 10 eV. To compare with the relatively hot plasma sheet ions, Figure 13d shows the distribution of hot plasma sheet ion temperatures coincidentally observed with FAL ion events, which are typically 3.4±0.8 keV with a median value of 3.3 keV. These values lead to ratios between cold FAL ions and hot plasma sheet ion temperatures that are found to be $4.2_{-1.5}^{+2.4}\times 10^{-3}$, with a median value of $4\times 10^{-3}$. Similarly, Figure 13h shows that hot plasma sheet ions coincident with transverse ions are associated with a little broader range of temperatures with values typically 3.7±1.1 keV. These give rise to typical cold transverse ion to hot plasma sheet ion temperature ratios of $2.8_{-1.4}^{+2.9}\times 10^{-3}$. In addition characteristic energies for FAL and transverse ions were also computed (not plotted). FAL ion characteristic energies range from 7 to 35 eV with a mean of 16 eV. Characteristic energies of transverse ions reach higher values generally ranging from 4 to 70 eV with a mean of 16 eV, with some values reaching ≳100 eV (0.1%) owing to enhanced convection.

**Figure 14:**
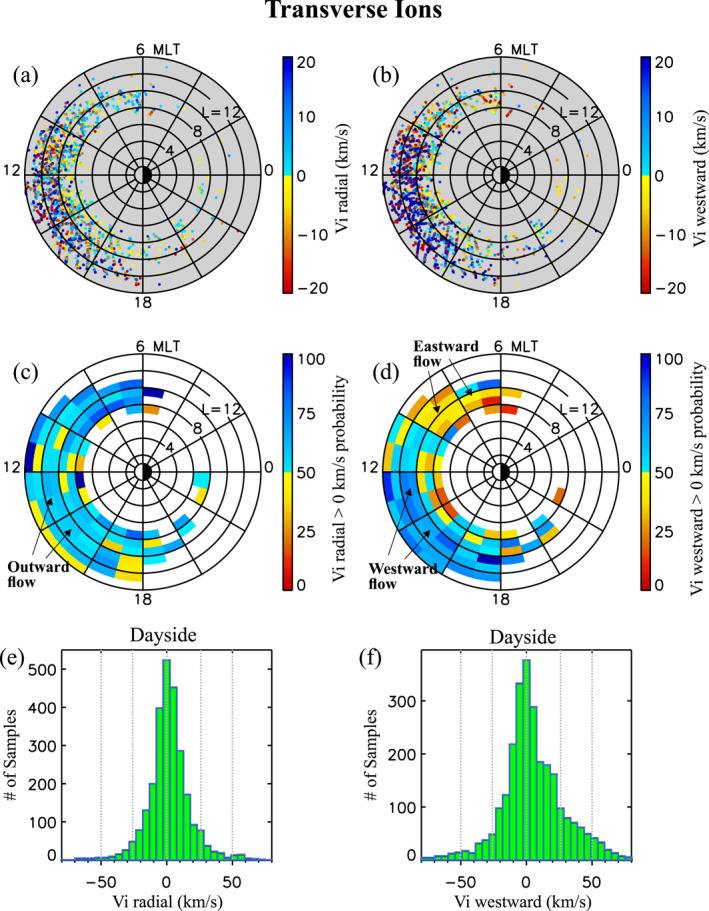
(a) Radial and (b) Westward velocity components of dense low energy, transverse ions as a function of L shell and magnetic local time (with noon to the left). Also shows the percentage of events with positive (c) Radial and (d) Westward velocities, and histograms of the (e) Radial and (f) Westward velocity components in the dayside. Note that only bins with three or more points are shown in panels (c) and (d).

To examine the bulk flow characteristics of cold dense transverse ions, Figures 14a and 14b display their radial and westward velocity components as a function of L shell and MLT (with noon to the left). The velocities shown were corrected for spacecraft motion. The velocities are color coded according to their magnitude and the positive and negative sense, with radial outward (inward) velocities corresponding to the cyan to blue (yellow to red) dots, and westward (eastward) velocities corresponding to cyan to blue (yellow to red) dots, respectively. Though there are exceptions, close inspection reveals that the transverse ion events are predominantly moving in the westward direction (blue in Figure 14b) from dusk and into the prenoon sectors with a tendency toward the magnetopause. To better illustrate this behavior, Figures 14c and 14d show the relative percentage of events with positive values of radial and westward velocities as a function of L shell and MLT, respectively. Percentages above 50% correspond to preferential outward (westward) flows, while values below 50% correspond to preferential inward (eastward) flows. The prominent cyan to blue color in Figure 14c reveals the preferential radially outward propagation tendency of these ions for most L shell and MLT values they occur. This radial tendency is for the most part slight with percentages ranging from just above 50%–65%. The strong preferential westward flow from dusk and into the prenoon sector is readily discernible in Figure 14d, with percentages reaching 70%–85%. However, at 6–10 MLT the flow tendency is reversed being predominantly eastward as indicated by the yellow to red color. In addition the bulk velocities in Figures 14a and 14b are shown to have larger values with increasing distance from Earth in the prenoon to dusk sectors, as indicated by the transition from cyan/yellow to darker blue and red colors. The distribution of radial and westward velocities in the dayside (6–18 MLT) are shown in Figures 14e and 14f, respectively. The distributions confirm the flow trends revealed via their preferential positive skews, with the radial skews being slight, while the westward skew being significant owing to the larger westward flow values reaching up to several tens of km/s.

**Figure 15:**
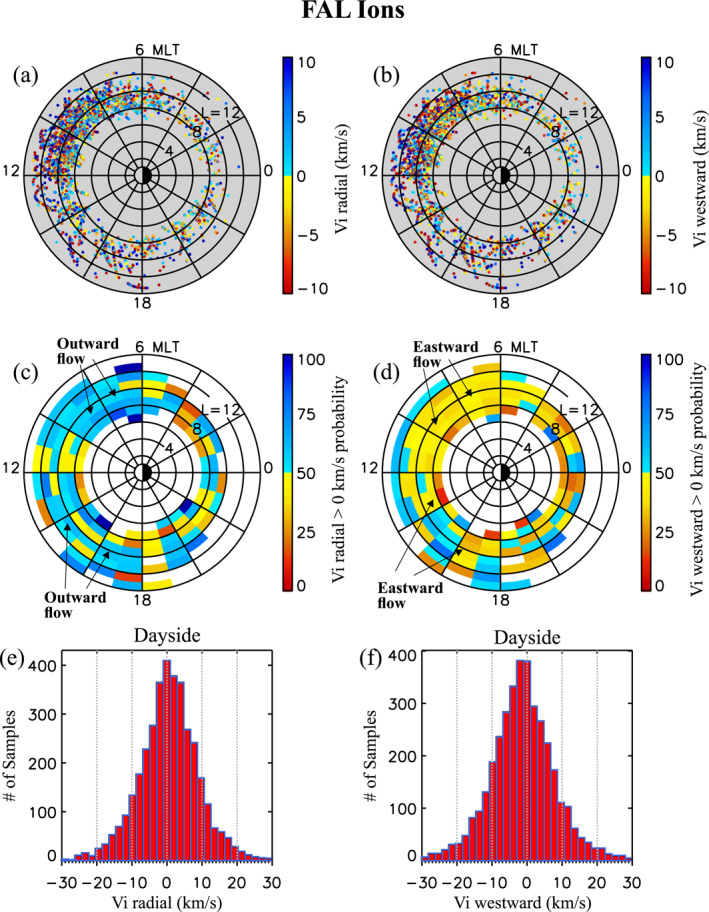
(a) Radial and (b) Westward velocity components of low energy, field‐aligned ions, and the percentage of events with positive (c) Radial and (d) Westward velocities as a function of L shell and magnetic local time (with noon to the left). Also shows histograms of the (e) Radial and (f) Westward velocity components in the dayside. Note that only bins with three or more points are shown in panels (c) and (d).

To examine the bulk flow characteristics of cold dense FAL ions, Figures 15a and 15b display their radial and westward velocity components (in an Earth fixed frame) as a function of L shell and MLT (with noon to the left). Also shown in Figures 15c–15f are the percentage of events with positive radial and westward velocities, and histograms of the radial and westward velocity components in the dayside, respectively. These results show that the FAL ions are associated with a lower flow spread than the transverse ions, with values being confined to <30 km/s. Also, in contrast to the transverse ions, the field‐aligned ions have a clear preferential eastward orientation, as indicated by the preponderance of yellow to red at all MLTs in Figure 15b, the yellow to orange in Figure 15d, and the peak/skew in the histogram of dayside westward velocities in Figure 15f. The radial velocities have a slight tendency to be oriented away from Earth toward the magnetopause, as indicated by the preponderance of cyan to blue in Figure 15c and also the slight positive skew in the distribution of radial velocities in Figure 15e.

The statistical differences in the radial and azimuthal flow of the two classes of cold low energy ions in the equatorial plane may be qualitatively explained as being the net result of the competition between convection and corotational electric fields (Kavanagh et al., 1968), but affected by the presence of the magnetopause in the dayside magnetosphere. Additional factors include the fact that the two classes arise from different sources and locations, and also different IMF $B_{z}$ and magnetospheric activity conditions. The cold transverse ions preferentially occur in the noon‐to dusk sector under southward IMF and during moderate to active magnetosphere conditions. Such conditions are associated with enhanced sunward magnetospheric convective flows that predominate over the counterclockwise drifts due to the corotational electric field over a larger extent of the magnetosphere. The effect of the enhanced convection on the duskside is to peel off and propel plasmaspheric ions in the bulge region in the sunward direction out to larger radial distances toward the magnetopause. A model that qualitatively demonstrates the effects of enhancements in the dawn‐dusk convection electric field in the magnetosphere is described by Grebowsky (1970). The preferentially larger azimuthal flows that extend into local noon shown in Figure 14b may be due to time dependent convection effects, such as during sudden sharp increases in convection acting on a pre‐existing plume, which can have the effect of propelling the plume further toward the subsolar magnetopause. It may also be the result of flows being diverted by the magnetopause boundary and entrained in westward oriented transverse convective flow along its surface toward the subsolar point, where they can participate and influence reconnection processes occurring therein (e.g., André et al., 2016; Borovsky et al., 2013; Walsh et al., 2014). Examples of dense plume ions observed in association with large tangential/westward flows along the magnetopause have been reported (e.g., McFadden et al., 2008b). Our results show that the preferential westward azimuthal flow occurs over a broad MLT extent at L≥10. The MLT spread of the azimuthal flows of these transverse ions is likely dependent on magnetospheric activity and enhanced solar wind pressure, as suggested in the occurrences of these ions in Figures 9 and 10. The azimuthal flows may also depend on IMF $B_{y}$, as suggested in previous studies (e.g., Chen & Moore, 2004). A detailed assessment of these effects on the equatorial flow characteristics is beyond the scope of this study and left for future work.

Though they tend to occur in the dusk sector, plume‐like ions have been observed at all MLT sectors including the dawn sector in CRRES satellite observations closer to Earth (Moldwin et al., 2004). Here we show that transverse ions having characteristics of plumes in the interval from 6 to 10 MLT have reversed easterly flow. This flow tendency is also attributable to the combined effects of enhanced sunward convection and corotational effects which are additive in this sector.

In contrast, the FAL ions occur under northward IMF during relatively quieter conditions. Under strong northward IMF $B_{z}$ conditions associated with lobe reconnection, it is expected that there should be an inward radial flow preference near noon (Chen & Moore, 2004). However, most of the events are characterized as having significant IMF $B_{y}$, in addition to IMF $B_{z}$ northward (see Figure 11b). Under weaker northward IMF conditions, reconnection with closed field‐lines and gross‐sunward convective flow transport can still happen. In either case corotational electric forces may play a more important role as convection in the equatorial plane is significantly reduced. This explains the reduced overall flow, and eastward preference revealed in Figure 15. The eastward preference is seen at all MLTs, including the enhanced FAL populations in the dusk‐sector. A similar behavior is manifested in the observed flow/electric field patterns under northward IMF at distances of 4<L<10 in the equatorial magnetosphere reported in the study by Matsui et al. (2003). In that study, the flow/electric field topology was determined from perpendicular electric fields measured by electron drift instrument on Cluster. Their results indicate that, while the corotational electric fields were largest at lower L values, these fields, though significantly diminished, were still influencing particle orbits at the largest distances of their statistical sample.

## 5 Summary and Conclusions

We examined the properties of cold dense low‐energy ions within Earth's outer magnetosphere (i.e., at geocentric distances >6$R_{E}$) and their relation to solar wind and magnetospheric conditions. We showed that cold dense low‐energy ions are pervasive in the magnetosphere during and/or after elevated solar wind dynamic pressure and/or active magnetospheric conditions. While observed at all MLTs, they are most prominent in the dayside magnetosphere, occurring over large azimuthal and radial (1–5 $R_{E}$) extents of most transects by the THEMIS spacecraft at distances from 6 to 14 $R_{E}$. The duration of such cold dense plasma in the magnetosphere is correlated with the Kp and/or solar wind pressure variations, with elevated densities lasting from a few days to a significant fraction of a month on gross time scales or few hours to a significant fraction of a day on shorter time variations. The cold dense ions typically have peak energies at few tens of eV and below with some exceptions reaching ∼100 eV associated with elevated flow near the magnetopause. These typically have densities of ∼1–2 ${cm}^{-3}$, with some examples reaching a few tens of ${cm}^{-3}$. These ions dominate the density when they occur yielding cold low‐energy ion to hot plasma sheet ion density ratios ranging from 2 to 70, with a mean value of ∼5. These ions are relatively cold with temperatures ranging from a few to tens of eV, with a mean of 13 eV. These ions are accompanied by cold (typically 6 eV) dense electrons at 0–25 eV energies.

The cold dense ions have distribution/flux characteristics that can be categorized as being convection dominated (transverse to the magnetic field), magnetic field‐aligned (uni‐ or bi‐directional), or combinations of both. The transverse/convection dominated ion events were shown to preferentially occur in the postnoon MLT sector of the magnetosphere with peak occurrence at ∼1330 MLT at L values from 8 to 14. These tend to occur during sustained moderate to strongly enhanced magnetospheric conditions under enhanced solar wind dynamic pressure. Dayside transverse ion occurrences were also shown to be sensitive to IMF clock angle, being depressed at purely northward IMF and peaking for large values of southward IMF $B_{z}$ orientations. IMF $B_{y}$ was also shown to impact transverse ion occurrences with a slight preference to larger westward orientations. The transverse ions were shown to have densities ranging from our imposed lower limit of 0.5 to tens of ${cm}^{-3}$, with a mean of 1.7 ${cm}^{-3}$. Comparisons reveal that the dayside transverse ion densities are positively correlated with solar wind dynamic pressure. Temperatures of these ions were shown to range from a few to tens of eV, with typical values of 11±6 eV. These ions are associated with a broad range of velocities, with larger values approaching several tens of km/s at larger radial distances in the postnoon sector. The velocities were shown to be preferentially westward directed with a slight preference toward the magnetopause indicating that these ions are radially expanding as they further penetrate into the dayside magnetosphere. Based on their preferential location in the dusk magnetosphere, low energy in the plasma frame, and westward flow tendency, these ions have characteristics of plasmaspheric plumes. The preferential westward drift from dusk to noon is consistent with the scenario that the equatorial drift paths of these ions are controlled by magnetospheric convection characteristic of active southward IMF conditions. These flows may also be affected by the magnetopause boundary, time dependent convection effects, and IMF $B_{y}$. The correspondences with solar wind dynamic pressure suggest that it is an important controlling factor of transverse ion densities and occurrences in the outer magnetosphere. This may be achieved through compression and enhanced global magnetospheric convection, respectively.

Dense low‐energy FAL ions are seen at all MLTs, but predominantly occur in the prenoon sector with peak occurrence at ∼0930 MLT at L values from 8 to 12. We showed that these tend to occur during quieter periods and under preferentially northward IMF orientations that occur within several hours after moderate to strongly active magnetospheric conditions. The occurrences of these ions were also shown to be correlated with solar wind dynamic pressure. However, unlike the plume‐like events, the FAL ion occurrences were shown to plateau (or increase slowly) after an initial sudden increase with pressure. The FAL ions have densities ranging from our imposed lower limit of 0.5 to ∼10 ${cm}^{-3}$, with a mean of 1.0 ${cm}^{-3}$. The dayside FAL ion densities were also shown to be positively correlated with solar wind dynamic pressure. Temperatures were shown to range from 5 to tens of eV, with typical values of 14±4 eV. The FAL ions are associated with relatively lower bulk velocities having values <30 km/s, with an eastward and preferentially outward flow tendency in the equatorial plane. Based on their dawnside location and eastward flow tendency these ions have characteristics of warm plasma cloak ions, but also include other ion outflow type populations. However, the characteristic energies of the FAL ions (several tens of eV and below, with a mean of 16 eV) are on the lower end of the energy scale for warm ion cloak. The overall reduced and eastward flows of these ions are consistent with their equatorial drift paths being controlled by corotational electric forces over the reduced overall magnetospheric convection that arises from the weaker northward IMF $B_{z}$ conditions these ions occur under. The preferential occurrence during northward IMF under quiet periods following moderate to strongly active magnetospheric conditions places important new constraints for models of the source of these ions, which currently presume steady state conditions and southward IMF orientations. The correspondences with solar wind dynamic pressure also indicate that it is an important controlling factor of the density and occurrence of FAL ions in the equatorial plane. This may be by increasing the FAL ion ionospheric source outflow population by enhancing soft electron precipitation and/or the Poynting flux into/Joule heating in the ionosphere. The fact that the dawnside bias in the low‐latitude/equatorial occurrences of the FAL ions shows remarkable similarity to that found for dayside upwelling ions observed in high‐latitude measurements from low‐altitude polar orbiting spacecraft reported in the literature (e.g., Lockwood et al., 1985; Redmon et al., 2012) suggests that these may be a significant, if not dominant, source population of the FAL ions reported herein.

## Data Availability

The data from the THEMIS mission used in this study are publicly available at the http://themis.ssl.berkeley.edu. OMNI plasma and field data from upstream solar wind monitors (i.e., Wind and ACE), and activity measures are available from https://omniweb.gsfc.nasa.gov/.
